# Study of Mono and Di-*O*-caffeoylquinic Acid Isomers in *Acmella oleracea* Extracts by HPLC-MS/MS and Application of Linear Equation of Deconvolution Analysis Algorithm for Their Characterization

**DOI:** 10.3390/ph16101375

**Published:** 2023-09-28

**Authors:** Maria Bellumori, Marco Pallecchi, Beatrice Zonfrillo, Luigi Lucio, Marta Menicatti, Marzia Innocenti, Nadia Mulinacci, Gianluca Bartolucci

**Affiliations:** NEUROFARBA Department, Section of Pharmaceutical and Nutraceutical Sciences, University of Florence, Via U. Schiff 6, Sesto Fiorentino, 50019 Firenze, Italy; marco.pallecchi@unifi.it (M.P.); beatrice.zonfrillo@unifi.it (B.Z.); luigi.lucio@unifi.it (L.L.); marta.menicatti@unifi.it (M.M.); marzia.innocenti@unifi.it (M.I.); nadia.mulinacci@unifi.it (N.M.)

**Keywords:** chlorogenic acids, tandem mass spectrometry, ion trap MS analyzer, isomers recognition, ERMS

## Abstract

Chlorogenic acids, the esters of caffeic and quinic acids, are the main phenolic acids detected in *Acmella oleracea* extracts and have gained increasing interest in recent years due to their important biological activities. Given their structural similarity and instability, the correct analysis and identification of these compounds in plants is challenging. This study aimed to propose a simple and rapid determination of the *A. oleracea* caffeoylquinic isomers, applying an HPLC-MS/MS method supported by a mathematical algorithm (Linear Equation of Deconvolution Analysis (LEDA)). The three mono- and the three di-caffeoylquinic acids in roots of *Acmella* plants were studied by an ion trap MS analyzer. A separation by a conventional chromatographic method was firstly performed and an MS/MS characterization by energetic dimension of collision-induced dissociation mechanism was carried out. The analyses were then replicated using a short HPLC column and a fast elution gradient (ten minutes). Each acquired MS/MS data were processed by LEDA algorithm which allowed to assign a relative abundance in the reference ion signal to each isomer present. Quantitative results showed no significant differences between the two chromatographic systems proposed, proving that the use of LEDA algorithm allowed the distinction of the six isomers in a quarter of the time.

## 1. Introduction

One of the most distinguished members of the genus *Acmella* is *Acmella oleracea* (L.) R.K. Jansen, an annual herb native to Brazil that is occurring throughout tropical and subtropical regions around the world. It has been widely cultivated for horticultural, medicinal, insecticidal, and culinary purposes, and recently, multidisciplinary studies on this herb have been promoted, with an increasing number of commercial products appearing on the market as personal care, health care, and culinary products [1].

Different extracts of *A. oleracea* have been reported to hold numerous important biological activities, including a local anesthetic property, which is the main reason why *A. oleracea* has been used since ancient times to relieve toothache. In addition, anti-inflammatory, analgesic, cytotoxic, antioxidant, antimicrobial, anthelmintic, antiwrinkling, aphrodisiac, and insecticidal/acaricidal properties are reported [1,2,3,4,5,6,7].

**Figure 1:**
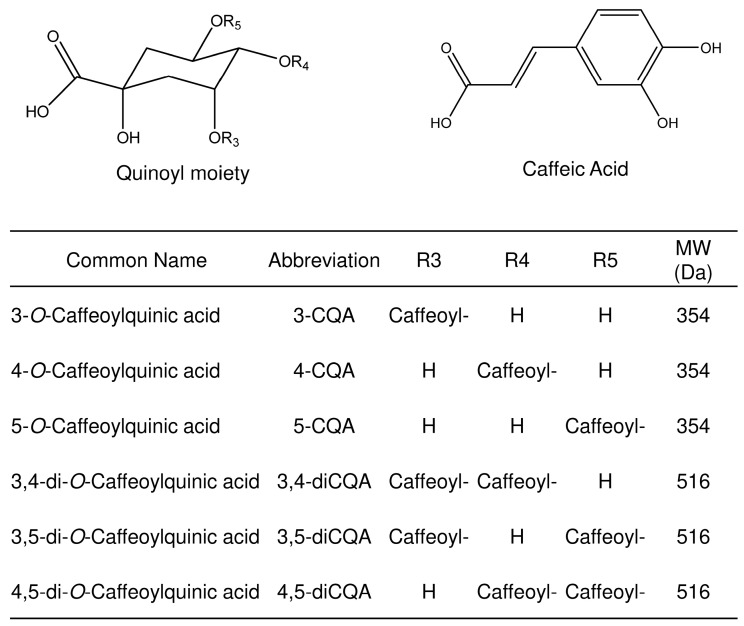
General structures, common names, abbreviations, and molecular weights of the studied analytes.

The richness and variety of secondary metabolites are mainly responsible for *A. oleracea* bioactivities. Nascimento et al. (2020) and Bellumori et al. (2022) [8,9] characterized many bioctive compounds, including mainly phenolic acids, glycosylated flavonoids, alkamides, and fatty acids, some of which specific to certain anatomical parts of the plant or cultivation system [10,11]. Of particular interest are the chlorogenic acids, a class of esters between caffeic and quinic acids which represent the main phenolic acids detected in this plant. These isomer compounds of *Acmella*, also found in many other plant species, are biologically important and have gained increasing interest in recent years [12,13,14]. However, due to their structural similarity and instability, and the fact that the number of commercially available standards is limited to just a few, the correct analysis and identification of these compounds in plants is a challenge. The most widespread analytical approach to solve this problem involves the chromatographic separation of the component of sample extract and their characterization by ultraviolet-visible (UV-Vis), mass spectrometry (MS), or tandem mass spectrometry (MS/MS) spectra [15,16,17]. Nevertheless, the application of this strategy often requires the set up of ad hoc sample preparation and the evaluation of different chromatographic columns or mobile phase composition to obtain adequate analytes separation. All these procedures are usually molecule-specific and rarely can be extended to other compounds. From this point of view, the application of MS detection can be a promising technology as it combines both the “universal” revelation features and compounds distinction proprieties, separating the analyte-specific ion component from the total acquired signal. Unfortunately, the MS-specific revelation capability find a strong limitation in the isomers analysis, when their characteristics lead to a common ion signals. However, numerous strategies have been proposed to solve the problem and some of these have involved the study of MS/MS features allowing the characterization and quantification of isomers and/or isobars in mixtures via a standardized approach, applicable to different compounds [18,19,20,21,22,23,24,25,26,27,28]. Our proposal aims to exploit the separation characteristics of mass spectrometry, reversing the guarantees of specificity of the analytical determination from the chromatographic system to the mass detector. We addressed this topic by introducing and developing Linear Equation of Deconvolution Analysis (LEDA), a mathematical tool that allows the recognition of isomers by processing MS/MS data without their chromatographic separation [29,30,31,32]. In this study an HPLC-MS/MS method supported by LEDA was applied in determination of the three caffeoylquinic acids (CQAs) and three di-caffeoylquinic acids (diCQAs) in *A. oleracea* (Figure 1). 

The introduced HPLC approach significantly simplifies the operating parameters, using a short column and a fast elution gradient. Following these arrangements, the chromatographic column was used only to avoid or limit the interference towards the analytes’ ionization process by the sample matrix (matrix effects), allowing the extension to recognition of other compounds present in the sample only modifying the MS/MS conditions [31,32].

The aim was to propose a simple and rapid determination and differentiation of the *A. oleracea* caffeoylquinic acid isomers (Figure 1), resolving the MS/MS spectra and assigning the correct signal to each isomer, even given chromatographically unresolved peaks. A single chromatographic set up was used, tuned to minimize the run time without requiring high efficiency or resolution between analytes, exploiting the distinguishing capabilities of MS/MS experiments. 

## 2. Results and Discussion

Recognition of isomers by applying the LEDA approach involves using a multistage MS system and collecting as much information as possible on the MS/MS behavior of each analyte. Regarding the MS system, in this study an ion trap (IT) MS analyzer was used that shows a peculiarity compared with other multistage instruments: the MS/MS experiments are performed in the same site, using time to modify the conditions applied on studied ions [33]. Then, a sequence of time-dependent steps was carried out to perform the selection of precursor ion, its fragmentation by collision-induced dissociation (CID) mechanism and detection of formed product ions (Pis) [34,35]. Thus, the sum of these time-steps leads to a longer MS/MS acquisition cycle in the IT compared with other multistage instruments, reducing the collection frequency of sample data collection and affecting its chromatographic profile. However, IT instruments show some advantages in MS/MS analysis, such as the sensitivity of the Pis scan acquisition (complete MS/MS spectrum), the different management of excitation energy, and the reiteration on a selected product ion of another tandem MS experiment (MS^n^) [36]. Concerning the collection of MS/MS data for each studied compounds, unfortunately, a complete panel of pure standards was not available in our lab. Therefore, the required information was gathered from the analysis of *Acmella* working solution (Acmella WS) through a conventional chromatographic separation of each caffeoylquinic-isomer and following its MS/MS characterization. Taking this information into account, we planned a series of studies to acquire the MS/MS data of each analyte, investigate the IT features, and define the most suitable MS/MS parameters for the isomers recognition using the LEDA approach.

The information collected for each caffeoylquinic-isomer present in Acmella WS was as follows:

- Separation by a conventional chromatographic method;
- MS/MS characterization by energetic dimension of CID mechanism;
- Interpretation of obtained MS/MS spectra (CID study);
- Application of the LEDA approach in Acmella WS;
- Assessment of the LEDA quantitative performances.

### 2.1. Conventional Chromatographic Separation of Caffeoylquinic-Isomers in Acmella WS

The complete separation of each studied analyte was necessary as their pure standards were not available in our lab. Hence, the separative capabilities of HPLC were exploited to “purify” the analyte signals, allowing a study to evaluate their individual MS/MS behavior. 

**Figure 2:**
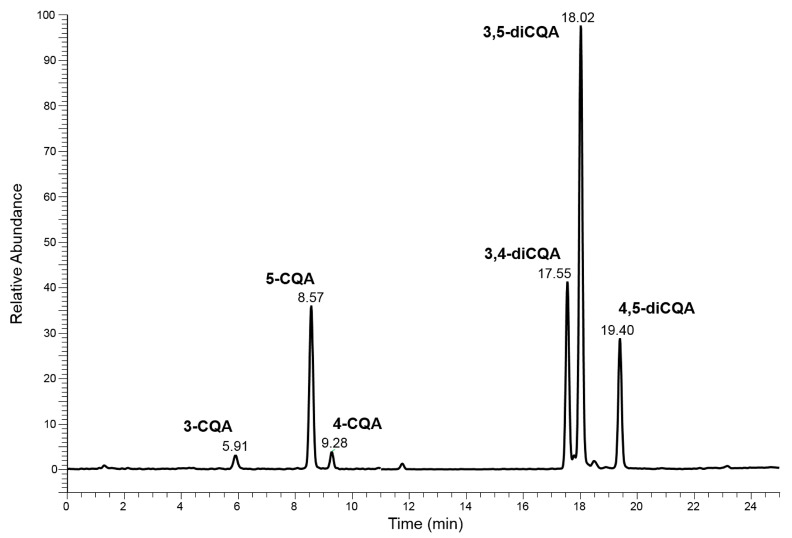
Chromatographic profile of the HPLC-MS analysis (ChromSys 1) of the negative ions in *m*/*z* range between 150 and 750 of the Acmella WS.

This purpose was achieved by using a conventional HPLC-MS approach which involved the use of a lengthy column, a slow elution gradient program and a long run-time (Chromatography system 1 or ChromSys 1, see Section 3.4). Considering only the peaks over 1% of relative abundance, the obtained chromatographic profiles showed two groups of isomers: the CQAs (3-, 5- and 4-CQA), characterized by [M-H]^−^ ion species at 353 *m*/*z* and the diCQAs (3,4-, 3,5- and 4,5-diCQA) that exhibit the [M-H]^−^ species at 515 *m*/*z*. The identification of the 5-CQA was achieved by correlation between the retention times and MS/MS spectra obtained with the pure standard, while the preliminary attribution of the other CQAs and diCQAs compounds were carried out by comparing the MS/MS spectra of unknown peaks with those published by M.N. Clifford et al. [37,38]. The proposed separation was found to be suitable for distinguishing each group of isomers, allowing the use of an appropriate MS/MS event, distributed over two different time segments. Figure 2 shows an example of the chromatographic profile of the HPLC-MS analysis, carried out with ChromSys 1, of the negative ions acquired in *m*/*z* range between 150 and 750 of the Acmella WS.

In the Supplementary Materials (Table S1) the calculated peak parameters (i.e., retention times, peaks width, efficiency, etc.) for each analyte have been reported. The resolution value (≥1.5) calculated for each contiguous pair of analytes assured the “purity” of every isomer peak, avoiding mutual interferences in following MS/MS characterization.

### 2.2. MS/MS Characterization by Energetic Dimension of CID Mechanism

**Figure 3:**
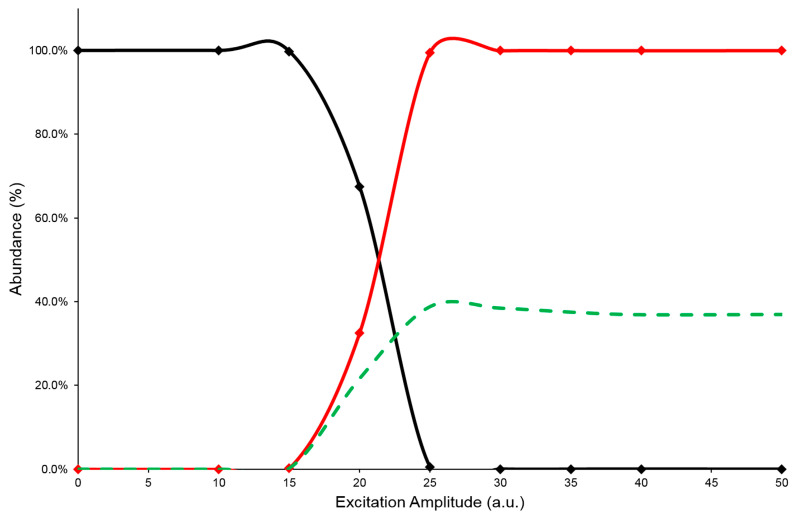
SY (black line), PiF (red line), and PiY (green dashed line) curves of the 5-CQA isomer at ExT 50 ms.

In the IT MS/MS experiment, the precursor ion is energized by applying an excitation amplitude (ExA) for an established time (excitation time or ExT) in order to raise the number of collisions with the helium gas, normally present inside the Its. The combination of ExA and ExT values allows the activation of the fragmentation channels of the precursor ion and the formation of the Pis. As reported above, pure standards for each component studied were not available; therefore, in order to obtain information on their CID behavior, a series of HPLC-MS/MS analyses were performed on [M-H]^−^ ion species of every caffeoylquinic-isomer of Acmella WS. Each analysis was carried out with the same chromatographic separation, using ChromSys 1, but different precursor ion energization. In a previous work, it was verified that the energization of the precursor ion during the CID process reached an equilibrium for the ExT values between 50 and 100 milliseconds (ms) [39]. Therefore, due to the long time for each HPLC-MS/MS analysis, the ExA values were varied in the range of 0 to 50 arbitrary units (a.u.), while the ExT value was kept constant at 50 ms. In this way, an energy resolved mass spectrometry experiment (ERMS) on each caffeoylquinic-isomer present in Acmella WS was simulated and the MS/MS data collected were used to evaluate the energetic dimension of CID process. This evaluation was performed by plotting the survival yield curves of precursor ion (SY), the Pis formation (PiF) and the Pis yield (PiY), as detailed in Section 3.3). A typical graph used to describe the CID energetic study was reported in Figure 3, while the graphs of the other isomeric compounds can be found in the Supplementary Materials (Figures S1–S5). 

The PiF curve (red line) represents the ratio between the sum of the intensities of Pis and the total abundance of detected ions (see Equation (2)). The PiF trend should describes the efficiency of CID process but the abundance ratios, used to build it, are not fully representative of the mechanism. Indeed, these ratio values neglect the signal loss due to the precursor ion ejection, during the excitation process, or caused by undetected product ions, because their *m*/*z* are below the low mass cut-off of the IT MS/MS experiment. As a result, the calculated PiF values often suffer from overestimation, depicting an incorrect CID efficiency. Therefore, to evaluate a reliable value of fragmentation yield from the CID process, the sum of the abundances of Pis was referred to the averaged intensity of the precursor ion before its fragmentation (PiY, see Equation (3)). By plotting the calculated values of PiY vs. ExA applied, a curve (green dashed line) is obtained that should best describe the efficiency of the CID process under the tested conditions. In this graph, the trend of the PiY curve increases achieving the highest value (PiYmax); the corresponding ExA value (ExAmax) represents the energy amplitude to be applied in the MS/MS process to reach the greatest abundance of Pis. Furthermore, observing the profiles of the SY plots, it is worth noting that the SY curve (black line) crosses the PiF curve (red line) at 50% abundance value: the related ExA value is characteristic for each compound subjected to the CID process and represents the ExA required to fragment 50% of the precursor ion (SY_ExA50_) [20,21]. In Table 1 are reported the data of SYExA50, PiYmax, and ExAmax obtained for the studied isomers.

The collected data described the energy requirement during the IT-CID process to fragment the precursor ion of the studied analytes. Most of the analytes showed an ExA_max_ of 25 a.u., with the exception of 3,4-diCQA, which required 30 a.u. to achieve the highest Pis formation. Therefore, in order to maintain the same CID condition for the two class of compounds (mono- and di-CQAs), the MS/MS time-event was set with an ExA value of 25 a.u. for the mono CQAs isomers, while an ExA value of 30 a.u. was used for the diCQAs isomers (see Section 3.5). It is worth noting that, for all the studied compounds, the PiY_max_ values were estimated to be around 50% (between 39% and 56%) compared with the abundance of the precursor ion, confirming a loss of ion signal during the MS/MS process. This decrease in signal was unavoidable and can be attributed to both the MS/MS mechanism used and the characteristics of the analyte.

### 2.3. Collision-Induced Dissociation Study

**Figure 4:**
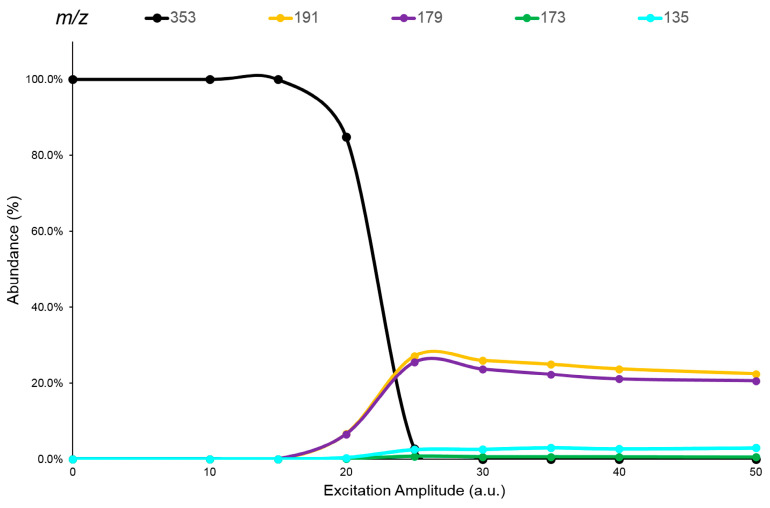
The breakdown curves of 3-CQA isomer obtained by elaboration of ERMS data. The legend reports each monitored ion (*m*/*z*) and its representative color in the graph.

The CQAs and diCQAs isomers studied (Figure 1), showed MS spectra in negative ions electrospray ionization (ESI) with only an abundant cluster signal representative of the deprotonated molecules ([M-H]^−^ species), with the same *m*/*z* value within the isomeric set (Supplementary Materials, Figures S6 and S7). Therefore, an ERMS study (see Section 3.4) was performed on all these isomers to enhance the differences between their fragmentation pathways and/or to find any specific Pis, which would allow them to be distinguished. The ERMS data of each analyte were processed and the collision breakdown curves were plotted; the obtained graphs were reported in Figure 4 and in the Supplementary Materials (Figures S8–S12). 

These graphs showed, after activation of their fragmentation channels, a constant Pis composition and the related formation curves followed the same trend achieving a highest value at the same ExA. The pattern of these breakdown curves demonstrates that the fragmentation channels of precursor ion in IT are simultaneously activated at a defined ExA. Hence, after complete dissociation of the precursor ion, the IT MS/MS spectra are the same despite the ExA increase; only PiY changed, reaching its maximum value at ExAmax. Concerning the fragmentation pathway of the analytes, M.N. Clifford et al. proposed an extensive study on the fragmentation behavior of CQAs and diCQAs. The CQAs isomers showed mainly three common negative ions: the quinic acid residue ion at 191 *m*/*z*, the caffeic acid ion at 179 *m*/*z* and a dehydrated quinic acid ion at 173 *m*/*z* [37]. The relative abundance of these ions differed between the considered isomers and can be useful for their distinction. Conversely, the diCQAs showed the formation of a favorite Pi at 353 *m*/*z* (relative abundance ≥ 25%), representative of the loss of a caffeoyl residue from the [M-H]^−^ species [38]. Other fragment ions were detected at low intensity (relative abundance ≤ 5%) which, together with the 353 *m*/*z* ion, can be useful for the isomer recognition. Indeed, all these ions showed different abundances depending on the origin precursor isomer. Therefore, the use of an appropriate ‘mathematical device’ suitable to distinguish the common signals by processing the MS/MS data and assigning the correct abundance to the isomer(s) present in the sample would be extremely useful. 

### 2.4. HPLC-MS/MS Supported by LEDA Approach

**Figure 5:**
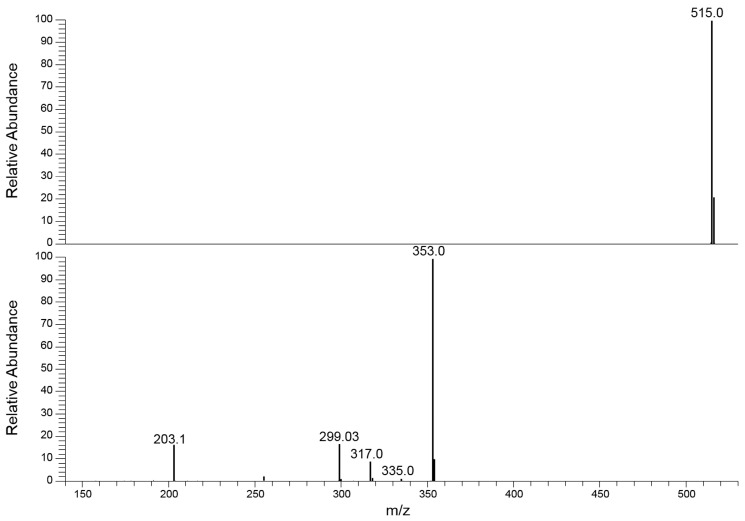
Comparison of the MS/MS spectra obtained at low ExA (MS/MS event 1, (**above**)), used to monitor the Ri signal, and at high ExA (MS/MS event 2, (**below**)), to acquire the Pis signals of 4,5-diCQA.

The LEDA post-processing mathematical device allows compounds to be distinguished in the analysis of complex samples by the elaboration of the acquired MS/MS data. The case of isomer recognitions is complicated by their similar characteristics such as the same elemental composition, molecular weight, and often common fragment ions. The novelty of the LEDA approach is based on the exploration of the “energetic dimension” MS/MS experiment to enhance possible intrinsic differences between the isomer compounds and allowing their recognition in a complex sample. This algorithm is based on the rationale that the MS/MS spectrum is represented as the sum of the contribution of each isomer present in the sample. As shown in the breakdown curves of Figure 4, each isomer produces the same panel of Pis with a different formation yield. To increase the reliability of these data and improve the significance value of differences on Pi yield, the abundance ratio of each Pi to a reference ion (Ri) was calculated. For this purpose, the Ri signal was chosen among the MS/MS signals having the same abundances, under given MS/MS conditions, among the studied isomers. Unfortunately, none of the Pis detected in MS/MS spectra of analytes had the required Ri features. However, in the analysis of isomers by the ESI source, it is reasonable to consider a similar behavior in their ionization process and then in the MS abundance signals (e.g., [M-H]^−^ species). Based on this hypothesis, the signal of the precursor ion was selected as Ri at the maximum intensity in the breakdown curve, without any fragmentation process occurring. In this way, the MS/MS experiment must be split into two events characterized by two different energetic levels (low and high) which allow to acquire both the unfragmented precursor ion (Ri) and its Pis spectrum. Also, by playing with “energetic dimension” of the MS/MS experiment, the acquisition of related ions (e.g., precursor and Pis) is permitted, monitoring the distinctive features between the isomers emphasized in the CID study. In this way, the ratio between the abundances of each Pi acquired and Ri represented the yield of formation of each Pi at selected ExA. In Figure 5 the comparison between the MS/MS spectra of 4,5-diCQA isomer obtained at low and high energetic level of MS/MS experiment is shown, while the corresponded MS/MS spectra related to other isomers were reported in Supplementary Materials (Figures S13–S17).

**Figure 6:**
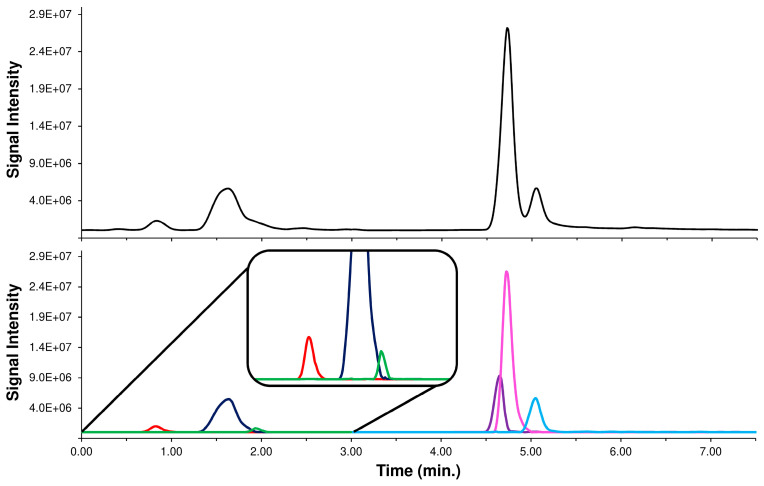
Chromatographic profile of the Ri signals (black lines) of Acmella WS with ChromSys 2 (**above**) and LEDA reconstructed chromatographic profiles of the 3-CQA (red line), 5-CQA (blue line), 4-CQA (green line), 3,4-diCQA (purple line), 3,5-diCQA (pink line), and 4,5-diCQA (light blue line) (**below**). A tenfold zoom of the abundance (inset) was reported to highlight the CGAs detected at low amount.

In an IT application, the use of the precursor ion signal as Ri requires inserting a low collision energy MS/MS event to enable acquisition of the unfragmented precursor ion (MS/MS event 1). Knowing the MS/MS behavior of each isomer, the LEDA tool elaborates each MS/MS ratio signal, distinguishes the possible components, and assigns the correct abundance to the isomers present. Profiting by LEDA features, it was possible to introduce a “standardized” methodological approach by simplifying the HPLC parameters, without worrying about the separation of the isomers [39]. To verify the proposed approach, we replicated the analysis of Acmella WS with a HPLC-MS/MS system assembled with a short column and using a fast elution gradient (see Section 3.5, Chromatography system 2 or ChromSys 2). The tuning of the elution parameters was carried out optimizing only the separation among CQAs and diCQAs isomers groups. This distinction was necessary to allow the setting up of the two MS/MS time segments, each dedicated to monitoring a set of isomers. Within each time segment, two MS/MS events are introduced to alternatively acquire the Ri and Pis signals (see Section 3.5). The proposed HPLC method, as expected, achieved only the partial components separation of the two major group of peaks, representative of the CQAs and diCQAs isomers. However, this result was reached in just ten minutes of chromatographic run without renouncing the solvents gradient elution, a desirable feature to avoid cross-contamination, due to unknown components, between the sample. To solve the partial coelution of the peaks and allow the distinction of the studied isomers, each acquired MS/MS data point was processed by the LEDA algorithm (‘scan-by-scan’ method) and a relative abundance in the Ri signal was assigned to each isomer present. Indeed, the abundance of Ri signal represents the sum of abundances of the precursor ions of all the isomers present in the elaborated data point. Hence, after the LEDA computing, a relative amount of Ri signal was allocated to each isomer present, with a consequent deconvolution of unresolved chromatographic peak (reconstructed chromatographic profile). Observing a typical reconstructed chromatographic profile, the performance of LEDA can be appreciated in the separation of co-eluted peak of the Ri signal that splits its abundance between the isomers, generating a reconstructed chromatographic trace of each recognized isomer. The comparison between a typical chromatogram obtained by analysis of Acmella WS with ChromSys 2 and its LEDA reconstructed chromatographic profiles are reported in Figure 6.

It should be noted that in the chromatogram reconstructed with LEDA, all the isomers studied (e.g., 5-CQA and 4-CQA or 3,4-diCQA and 3,5-diCQA) can be distinguished even under conditions of coelution or different relative abundance. Naturally, the reliability of LEDA in the signal assignment must be verified, by comparing the quantitative data obtained from ChromSys 2 with those obtained from conventional HPLC-MS/MS analysis (ChromSys 1).

### 2.5. Evaluation of Quali-Quantitative Performance of LEDA Approach

The lack of pure analyte standards did not allow us to prepare spiked samples, with known concentration and composition, to evaluate the analytical performance (accuracy and precision) of the LEDA approach. Therefore, the same sample of Acmella WS were independently analyzed by the two proposed chromatographic systems and the results of quali-quantitative analyses of CQAs and diCQAs were compared. The determination of the CQAs and diCQAs in the conventional HPLC-MS/MS analysis (ChromSys 1) was performed by integration of every peak area on the Ri signal corresponding to the separated analytes (see Figure 2). Each integrated area was converted in quantitative data through the external standard method by referring to a five levels calibration curve built analyzing standard solutions of chlorogenic acid (5-CQA) with the same HPLC-MS/MS method. Also, for the 5-CQA, the Ri signal (unfragmented precursor ion) was used for the integration of peak areas. Indeed, as previously reported, these analytes have a different behavior during the CID process, with formation of Pis at different yields. Thus, using the unfragmented precursor ion (Ri) as the quantitative signal appeared to be the best compromise as reference for a quantitative evaluation. The analysis performed with chromatographic system 2 did not allow the quantitative estimation of the analytes, as the poor separation of the isomers prevented their recognition. Therefore, it was necessary to integrate all the MS/MS signals selected during the LEDA setup, calculate each Pi vs. Ri ratio, and process them with the proposed “mathematical device”. Also in this case, the Ri area was distributed among the isomers present in chromatographic peak, respecting their relative abundances (peak purity). The Ri assigned area of each recognized isomer was converted into quantitative data by referring to the same 5-CQA calibration curve previously used. The Acmella WS was analyzed six times for each proposed chromatographic system to allow evaluation of the precision of quantitative approaches by calculating the deviation standard (SD) of the results. Table 2 showed the quantitative data obtained by analyzing the Acmella WS with the conventional HPLC separation (ChromSys 1) and the new proposed LEDA approach (ChromSys 2). 

Quantitative results showed no significant differences between the ChromSys used, demonstrating that both the analytical approaches lead to the same results, albeit ChromSys 2 in a quarter of the time (Supplementary Materials, Figure S17). Particularly interesting were the discriminating properties of the LEDA algorithm in the determination of 4-CQA, estimated at a low concentration level in the Acmella WS. In fact, the 4-CQA in the ChromSys 2 was coeluted with 5-CQA, present at a ten times higher concentration in the sample; despite this quantitative significant difference, the LEDA effectively processed the MS/MS data distinguishing the correct amount of the 4-CQA signal from the peak tail of the 5-CQA (Figure 6, inset).

## 3. Materials and Methods

### 3.1. Chemicals and Instruments

Ultrapure water (resistivity 18 MΩ cm) was obtained by the Milli-Q-system Millipore (Milan, Italy). Acetonitrile, ethanol, *n*-hexane (Chromasolv), formic acid, ammonium formate (MS grade), and chlorogenic acid or 5-CQA (purity ≥ 95%) were purchased from Merck (Milan, Italy). The HPLC-MS/MS analysis was carried out by using a Thermo LCQ Deca XP plus ion trap (Waltham, MA, USA) equipped with Surveyor liquid chromatography system and an electrospray ion source (ESI). Raw data were collected and processed by Excalibur version 1.4 software.

### 3.2. Standard and Calibration Solutions

Stock solution of 5-CQA was prepared in acetonitrile at 1.0 mg mL^−1^ and stored at 4 °C. The 5-CQA working solutions were freshly prepared by diluting stock solutions up to a concentration of 100 mg L^−1^ and 10 mg L^−1^ (working solutions 1 and 2, respectively) in mixture of ultrapure water:acetonitrile 50:50 (*v*/*v*). The quantitative data of each analyte of Acmella WS were calculated by an external standard method, referring to the 5-CQA calibration curve. This curve was built analyzing five level calibration solutions prepared by adding a proper volume of working solution (1 or 2) and diluted up to 1 mL with ultrapure water:acetonitrile 50:50 (*v*/*v*) solution. Final concentrations of 5-CQA calibration solutions were: 1.0, 2.5, 5.0, 10.0, and 20.0 mg L^−1^, respectively. Each calibration solution was analyzed six times by the ChromSys 1 proposed method.

### 3.3. Preparation of Phenolic Extracts

The powdered roots obtained from in vitro seedling plants from regenerating lines derived by organogenesis of Acmella oleracea were extracted as reported in Bellumori et al. [9]. Briefly, 0.5 g were extracted twice with 10 mL of ethanol 80% (*v*/*v*) at 60 °C for 10 min under sonication and centrifuged at 5000 g for 10 min. The two supernatants were collected and defatted with n-hexane (1:2 *v*/*v*); the hydroalcoholic extract was then dried under vacuum at 35 °C, re-dissolved with exactly 5 mL of the same extractive mixture and stored a −20 °C (Acmella extract sample or Acmella ES). To perform the MS and MS/MS study, a diluted solution of the Acmella extract sample was daily prepared, collecting 50 μL and diluting up to 1 mL by ultrapure water:acetonitrile 50:50 (*v*/*v*) mixture (Acmella working solution or Acmella WS).

### 3.4. MS and ERMS Experiments

In all the reported MS and MS/MS analysis in this manuscript, the ESI source operates in negative ion mode by using the following setting: 5 kV source voltage, 45 arbitrary units (a.u.) sheath gas, 5 a.u. auxiliary gas, the capillary voltage was 25 V while its temperature was set at 280 °C, and 40 V of the tube lens. The isomers studied are structurally characterized by a free carboxylic function in position 1 of the quinoyl-moiety and therefore their dissociation as carboxylate ions in ESI source is favored. The MS analyzes, used to characterize the Acmella WS, were carried out in ions scan mode by monitoring the *m*/*z* range from 150 to 750 with 50 ms of maximum ion trap fill time. The MS/MS study, scheduled to study the energetics of the fragmentation of [M-H]^−^ species of each analyte and build its breakdown curves [39], were carried out increasing the ExA (named Normalized Collision Energy in the control panel of the instrument) stepwise in the range 0–50 a.u. To obtain the information about the fragmentation and CID behavior of each analyte, a series of HPLC-MS/MS analysis of the same Acmella WS was performed by using a common HPLC elution (ChromSys 1) with different MS/MS applied energies in CID process. The entire MS/MS analyzes batch represents the ERMS experiment and it allows the description of the energetic dimension of CID process. The other common parameters used for all the ERMS experiment were: 3 *m*/*z* of precursor isolation width, 50 ms of ExT, and 0.25 of q value. The obtained ERMS data were used to build the graphs that describe the energetic dimension of CID process (SY curves of precursor ion, the Pis formation, and the Pis yield). The SY curve describes the energetics of degradation of precursor ion according to grow of the ExA. Each SY value at defined ExA is calculated as follows:

(1) $$SY(\%)=\frac{Precursor\;ion\;Abundance}{Precursor\;ion\;Abundance+\sum_{1}^{n}Pis\;Abundances}\times 100$$

Likewise, the Pis formation (PiF) curve describes the energetics of formation of all Pis with the increment of applied ExA. Each PiF value at defined ExA is calculated as follows:

(2) $$PiF(\%)=\frac{\sum_{1}^{n}Pis\;Abundances}{Precursor\;ion\;Abundance+\sum_{1}^{n}Pis\;Abundances}\times 100$$

The Pis yield (PiY) is calculated to depict the efficiency of CID process and is estimated by ratio between the sum of abundances of the Pis at defined ExA vs. the average intensity of precursor ion before its decay. Normally, the higher abundance of precursor ion is observed at low ExA values, before the activation of fragmentation channels. The average of these abundances represents the quantity of precursor ion signal (Precursor ion max) available for CID process, while the sum of Pis abundances is the signal remained. Then, the yield of CID process should be described as follows:

(3) $$PiY(\%)=\frac{\sum_{1}^{n}Pis\;Abundances}{Precursor\;ion\;max}\times 100$$

Finally, the CID graphs were plotted to describe the dependence of fragmentation/formation processes (breakdown curves) from the energy provided to the precursor ion. The breakdown curves were plotted by reporting the ratio between the intensity peak values of each ion signal in the MS/MS spectra versus the precursor ion max at the ExA applied. In this way, the ratio of the abundances represents the yield of formation of each Pi respect to the precursor ion to the applied ExA.

### 3.5. HPLC-MS/MS Methods

In this manuscript two different chromatographic set ups were employed to achieve the planned aims. The first system, abbreviated as ChromSys 1, was used to separate each analyte in Acmella WS and was coupled with both MS and MS/MS detection mode. ChromSys 1 is distinguished by the use of a column Agilent Poroshell 120 EC-C18 2.1 × 150 mm, 2.7 mm particle size (Santa Clara, CA, USA), eluting with a gradient mobile phase. The program of elution gradient was set up as follows: initial at 95% solvent A, then decreased to 75% in 20.00 min, and to 10% in 3.25 min, kept at 10% for 9.75 min, returned to initial conditions in 0.01 min and maintained for 9.99 min to a total run time of 45.00 min. A second chromatographic system (ChromSys 2) was used to analyze the sample with the LEDA approach. The column used by this system was an Agilent Poroshell 120 EC-C18 2.1 × 30 mm, 2.7 mm particle size (Santa Clara, CA, USA), also employing a mobile phase elution gradient. This program of elution gradient was set up as follows: initial at 95% solvent A, then decreased to 70% in 5.00 min and to 10% in 0.10 min respectively, kept at 10% for 2.40 min, returned to initial conditions in 0.01 min and maintained for 2.49 min to a total run time of 10.00 min. The common parameters used by both chromatographic systems were: the mobile phase was maintained at constant flow of 0.25 mL min^−1^, column temperature at 40 °C and the injection volume was 5 µL. Finally, the used solvents were 10 mM formic acid in ultrapure water (solvent A) and 10 mM formic acid in acetonitrile (solvent B).

The elaboration of data from the ERMS experiment allowed the definition of the CID conditions used to compile the MS/MS methods. Indeed, each used chromatographic system was coupled by a specific MS/MS method that assured the correct application of CID parameters for the detection of the studied analytes. These conditions can be divided into common parameters, such as isolation width 3 *m*/*z*, 0.25 as q value, and ExT of 50 ms, or MS/MS event specifics, listed and reported in Table 3.

The MS/MS methods are both characterized by two acquisition time segments, each one split into two MS/MS events. The acquisition time segments allow the correct application of MS/MS events to CQAs or diCQAs isomers and their time range value depends on the by the chromatography system used. While the MS/MS events defined the acquisition of the ion signals that, alternately applied, allow the recording in ions scan mode of the Ri and Pis signals of the studied isomers. Both the HPLC-MS/MS methods were applied to analyze the Acmella WS, repeating the examination six times for each system to compare the quantitative results between the conventional and LEDA approaches.

### 3.6. The LEDA Alghorithm

The MS/MS spectrum of mixture of isomer or isobar compounds, after complete fragmentation of precursor ion, is represented by the sum of contribution of each component present in the mixture. As described above, the isomer compounds often produce, during MS/MS experiments, common Pis but with different yield of formation. To enhance the compound-dependent Pi yield differences and managing reliable data, the relative abundances of selected Pis with respect to Ri were calculated (see Section 3.6). Therefore, knowing the characteristic abundance ratios (Pi/Ri) of pure isomer, a deconvolution of acquired MS/MS signals is possible based on a series of linear regression equation as follows:

(4) $${\left(\frac{Pi}{Ri}\right)}_{m}=\sum_{x=1}^{n}{\left(\frac{Pi}{Ri}\right)}_{x}*{\left[\%\right]}_{x}$$

- (Pi/Ri)_m_ is the abundance ratio between the product ion (Pi) vs. reference ion (Ri) measured (m) in the sample;
- (Pi/Ri)_x_ is the characteristic abundance ratio between the Pi vs. Ri of pure isomer;
- [%]_x_ is the concentration (%) of the isomer in the sample.

Theoretically, considering a simple binary mixture of isomers (A–B), a single Equation (4) related to only a product ion ratio (Pi/Ri) could be sufficient. Indeed, by assuming that only the pair of isomers constitutes the MS/MS signal, the concentration of B is calculated as B% = (1 − A)%. However, in this case the possible contribution of signals from unknown isomers (or any co-eluting compound having the same precursor and product ions) is neglected. Therefore, the MS/MS signal processing “mathematical device” for recognizing mixtures of n isomers (LEDA algorithm), is preferable to be a matrix with at least n linear Equation (4). Naturally, to increase the specificity and reliability of isomers speciation, an overdetermined system of linear equations can be assembled; in this case the LEDA matrix was composed by a number > n of linear Equation (4). This is the case of IT that operates in MS/MS acquiring the Pis in a defined range of *m*/*z* (product ions scan); then, all Pis signals included in the range are recorded and can be used to set up the LEDA matrix. In detail, the proposed application leads to the determination of two isomers groups (CQAs and diCQA), acquired with distinct CID conditions and different precursor and product ions monitored, then two LEDA matrices were setup to elaborate the MS/MS data referring to each group of isomers. Considering only the Pi/Ri ratios ≥ 2%, two overdetermined system of linear equations can be assembled to solve the CQAs and diCQA isomers sets (supplementary materials, Equations (S1) and (S2)). A general equation to solve each overdetermined LEDA matrix was reported in supplementary materials (Equation (S3)). The characteristic abundance ratios were calculated by data obtained from ERMS experiment described above. The ratios between Pi vs. Ri selected in the MS/MS methods were calculated and the resulting values were reported in Table 4.

The deconvolution was performed by applying the algorithm either to the area abundances, obtained from the integrated peak intensities of each Pi signal, or to individual MS/MS data point of the chromatographic sample profile. In the first case, the LEDA provides the relative amounts (%) of each known component present in the sample. In the second approach, each MS/MS signal is deconvoluted ‘scan-by-scan’ and assigned to the present isomers, allowing a graphical separation of the processed chromatographic profiles. All calculations for the deconvolution of MS/MS data are processed using an Excel™ macro. 

### 3.7. Calibration Curve of HPLC-MS/MS Methods

The quantitative data on CQAs and diCQAs isomers, present in *Acmella* sample, were calculated by an external standard method referring on 5-CQA standard solutions. The HPLC-MS/MS analyzes with ChromSys 1 of the calibration solutions of 5-CQA were used to build its calibration curve, obtained by plotting the Ri peak area versus the nominal concentration of each calibration solution. A linear regression analysis was applied to obtain the best fitting function between the calibration points (Supplementary Materials, Figure S19). In order to obtain reliable LOD and LOQ values, the standard error (SE) of response and slope approach was employed [40]. The estimated SEs of responses of each analyte were obtained by the SE of y-intercepts (Y-SE) of regression lines elaboration of data obtained from the HPLC-MS/MS analysis of calibration solutions [31]. The results of 5-CQA calibration curve obtained for MS/MS Ri quantitation signal, defined as linear regressions parameters (slope and y-intercept), the determination coefficient (R^2^), and the estimated LOD and LOQ values for each analyte are reported in Supplementary Materials (Table S2). The Ri signal should be remembered that represents the precursor ion of the analyte not yet fragmented in the IT-MS/MS experiment; hence, its intensity depends by the molecule characteristics (e.g., weight, functional groups, ionization proprieties, etc.). Since the analytes are isomers or homologous derivatives, it is reasonable that their ionization yield inside the ESI source are similar and, consequently, their response factor (ratio between Ri abundance versus concentration). Then, this 5-CQA calibration curve represents the best compromise as quantitative reference for the determination of CQAs and diCQAs in *Acmella* sample. 

## 4. Conclusions

This study proposed a simple and rapid determination of the *A. oleracea* caffeoylquinic isomers, applying an HPLC-MS/MS method supported by LEDA algorithm. The three mono- and the three di-caffeoylquinic acids in roots of *Acmella* plants were characterized by LEDA which allowed to assign a relative abundance in the Ri signal to each isomer present. In fact, although ChromSys2 allowed to obtain only a partial separation of the isomers which did not permit their quantitative estimation, the processing of the MS/MS data with LEDA led to the deconvolution of the unresolved chromatographic peaks. This result was achieved in just ten minutes of chromatographic run, without renouncing the solvent elution gradient and allowing the distinction of the six isomers in a quarter of the time compared with conventional chromatographic methods.

The obtained results demonstrate the effectiveness of the LEDA algorithm in recognizing and separating the different isomers present in complex samples without the need of good chromatographic resolution.

## Figures and Tables

**Table 1:** The characteristic parameters of CID process such as efficiency (PiY_max_), SY_ExA50_ values, and related applied ExA (ExA_max_) calculated from ERMS experiments carried out on caffeoylquinic-isomers of Acmella WS. A.u., arbitrary units.

Compound	SY_ExA50_ (a.u.)	ExA_max_ (a.u.)	PiY_max_ (%)
3-CQA	21.0	25	56
5-CQA	20.5	25	53
4-CQA	19.9	25	39
3,4-diCQA	24.4	30	41
3,5-diCQA	18.1	25	39
4,5-diCQA	22.1	25	43

**Table 2:** Comparison between the quantitative data obtained by analyzing the Acmella WS with ChromSys 1 and 2.

Isomers	ChromSys 1 Acmella WS ± SD (mg L^−1^)	ChromSys 1 Acmella ES ± SD (mg L^−1^)	ChromSys 2 Acmella WS ± SD (mg L^−1^)	ChromSys 2 Acmella ES ± SD (mg L^−1^)
3-CQA	0.6 ± 0.1	12 ± 2	0.8 ± 0.1	15 ± 2
5-CQA	6.0 ± 0.5	120 ± 10	5.5 ± 0.2	110 ± 4
4-CQA	0.4 ± 0.1	8 ± 2	0.4 ± 0.1	8 ± 2
3,4-diCQA	6.5 ± 0.5	130 ± 10	7.0 ± 0.5	140 ± 10
3,5-diCQA	16.5 ± 1.0	330 ± 20	18.0 ± 0.5	360 ± 10
4,5-diCQA	4.0 ± 0.5	80 ± 10	4.5 ± 0.5	90 ± 10

**Table 3:** Time segments and MS/MS parameters used for acquisition of the CQAs and diCQAs isomers by using ChromSys 1 or 2.

	Time Segment	Time Segment (min)	Precursor Ion (*m*/*z*)	MS/MS Event	Pis Scan Range (*m*/*z*)	ExA (a.u.)
ChromSys 1						
CQAs	1	0.0–11.0	353	Ri	300–365	15
				Pis	95–250	25
diCQAs	2	11.0–25.0	515	Ri	450–530	10
				Pis	140–365	30
ChromSys 2						
CQAs	1	0.0–3.0	353	Ri	300–365	15
				Pis	95–250	25
diCQAs	2	3.0–7.5	515	Ri	450–530	10
				Pis	140–365	30

**Table 4:** Characteristic ion abundance ratios (Pi/Ri) ± standard deviation (SD) calculated by MS/MS data from 100 ng mL^−1^ solution of each pure isomer by ChromSys 1 described in Section 3.5.

Isomers Group	Ratio Pi/Ri (*m*/*z*)	3-CQA Ratio Value ± SD	5-CQA Ratio Value ± SD	4-CQA Ratio Value ± SD
CQAs	191/353	0.23 ± 0.01	0.30 ± 0.01	0.02 ± 0.01
	179/353	0.20 ± 0.01	0.02 ± 0.01	0.20 ± 0.01
	173/353	0.01 ± 0.01	0.01 ± 0.01	0.17 ± 0.01
	135/353	0.02 ± 0.01	0.01 ± 0.01	0.01 ±0.01
	**Ratio Pi/Ri** **(*m*/*z*)**	**3,4-diCQA** **Ratio Value ± SD**	**3,5-diCQA** **Ratio Value ± SD**	**4,5-diCQA** **Ratio Value ± SD**
diCQAs	353/515	0.28 ± 0.01	0.37 ± 0.01	0.26 ± 0.01
	335/515	0.02 ± 0.01	0.01 ± 0.01	0.01 ± 0.01
	317/515	0.01 ± 0.01	0.01 ± 0.01	0.02 ± 0.01
	299/515	0.02 ± 0.01	0.01 ± 0.01	0.04 ± 0.01
	203/515	0.02 ± 0.01	0.01 ± 0.01	0.04 ± 0.01

## Data Availability

Data is contained within the article.

## Supplementary Materials

The following supporting information can be downloaded at: https://www.mdpi.com/article/10.3390/ph16101375/s1, Table S1. Chromatographic parameters for each analyte obtained with the proposed HPLC-MS analysis; Table S2. Results of the calibration curve obtained for Ri signal of chlorogenic acid (or 5-CQA), defined as linear regressions parameters (slope and y-intercept), the determination coefficient (R^2^) and the estimated LOD and LOQ values; Figure S1. Precursor SY curve (black line), PiF (red line) and PiY (green dashed line) of the 3-CQA isomer at ExT 50 ms; Figure S2. Precursor SY curve (black line), PiF (red line) and PiY (green dashed line) of the 4-CQA isomer at ExT 50 ms; Figure S3. Precursor SY curve (black line), PiF (red line) and PiY (green dashed line) of the 3,4-diCQA isomer at ExT 50 ms; Figure S4. Precursor SY curve (black line), PiF (red line) and PiY (green dashed line) of the 3,5-diCQA isomer at ExT 50 ms; Figure S5. Four precursor SY curve (black line), PiF (red line) and PiY (green dashed line) of the 4,5-diCQA isomer at ExT 50 ms; Figure S6. ESI negative ions spectra of 3-CQA (upper), 5-CQA (middle) and 4-CQA (bottom); Figure S7. ESI negative ions spectra of 3,4-diCQA (upper), 3,5-diCQA (middle) and 4,5-diCQA (bottom); Figure S8. The breakdown curves of 5-CQA isomer obtained by elaboration of ERMS data; Figure S9. The breakdown curves of 4-CQA isomer obtained by elaboration of ERMS data; Figure S10. The breakdown curves of 3,4-diCQA isomer obtained by elaboration of ERMS data; Figure S11. The breakdown curves of 3,5-diCQA isomer obtained by elaboration of ERMS data; Figure S12. The breakdown curves of 4,5-diCQA isomer obtained by elaboration of ERMS data; Figure S13. Comparison of the MS/MS spectra obtained at low ExA (MS/MS event 1, above), used to monitor the Ri signal, and at high ExA (MS/MS event 2, below), to acquire the Pis signals of 3-CQA; Figure S14. Comparison of the MS/MS spectra obtained at low ExA (MS/MS event 1, above), used to monitor the Ri signal, and at high ExA (MS/MS event 2, below), to acquire the Pis signals of 5-CQA; Figure S15. Comparison of the MS/MS spectra obtained at low ExA (MS/MS event 1, above), used to monitor the Ri signal, and at high ExA (MS/MS event 2, below), to acquire the Pis signals of 4-CQA; Figure S16. Comparison of the MS/MS spectra obtained at low ExA (MS/MS event 1, above), used to monitor the Ri signal, and at high ExA (MS/MS event 2, below), to acquire the Pis signals of 3,4-diCQA; Figure S17. Comparison of the MS/MS spectra obtained at low ExA (MS/MS event 1, above), used to monitor the Ri signal, and at high ExA (MS/MS event 2, below), to acquire the Pis signals of 3,5-diCQA; Figure S18. Comparison between the HLPC-MS/MS analysis of Acmella WS with ChromSys 1 (above) and ChromSys 2 (bottom); Figure S19. The calibration curve of 5-CQA; LEDA algorithm, Equations (S1)–(S3).
