# Supplementary material for: Study of Mono and Di-O-caffeoylquinic Acid Isomers in Acmella oleracea Extracts by HPLC-MS/MS and Application of Linear Equation of Deconvolution Analysis Algorithm for Their Characterization
Source: Pharmaceuticals (Basel). 2023 Sep 28;16(10):1375. doi: 10.3390/ph16101375 (PMC10610532; doi:10.3390/ph16101375)
Supplement: Supplementary file 1 [file pharmaceuticals-16-01375-s001.zip › pharmaceuticals-2600899-supplementary.pdf]

Study of Mono and Di-*O*-caffeoylquinic Acid Isomers in *Acmella oleracea* Extracts by HPLC-MS/MS and Application of Linear Equation of Deconvolution Analysis Algorithm for Their Characterization

Maria Bellumori *, Marco Pallecchi, Beatrice Zonfrillo, Luigi Lucio, Marta Menicatti, Marzia Innocenti, Nadia Mulinacci and Gianluca Bartolucci *

Supplementary Materials

Table S1. Chromatographic parameters for each analyte obtained with the proposed HPLC-MS analysis. Rt, retention times ± error (2 standard deviation or 2 SD); Base Width, base peaks width; N, efficiency; R, resolution between contiguous analytes. The void time measured for the chromatographic system used was 1.02 min.

	Rt (min)	2 SD (min)	Base Width (min)	2 SD (min)	N	R
3-CQA	5.93	0.04	0.38	0.05	3895	-
5-CQA	8.58	0.04	0.43	0.06	6675	6.6
4-CQA	9.29	0.04	0.31	0.05	14358	2.2
3,4-diCQA	17.53	0.04	0.30	0.05	54601	-
3,5-diCQA	18.01	0.04	0.32	0.05	50680	1.6
4,5-diCQA	19.39	0.04	0.37	0.05	43955	4.0

Table S2. Results of the calibration curve obtained for Ri signal of chlorogenic acid (or 5-CQA), defined as linear regressions parameters (slope and y-intercept), the determination coefficient (R^2) and the estimated LOD and LOQ values.

Compound	MS/MS signal	Slope (counts/mg L^{-1})	y-Intercept counts)	R^2	LOD (mg L^{-1})	LOQ (mg L^{-1})
5-CQA	Ri	6.2 10^6	8.5 10^4	0.999	0.1	0.4

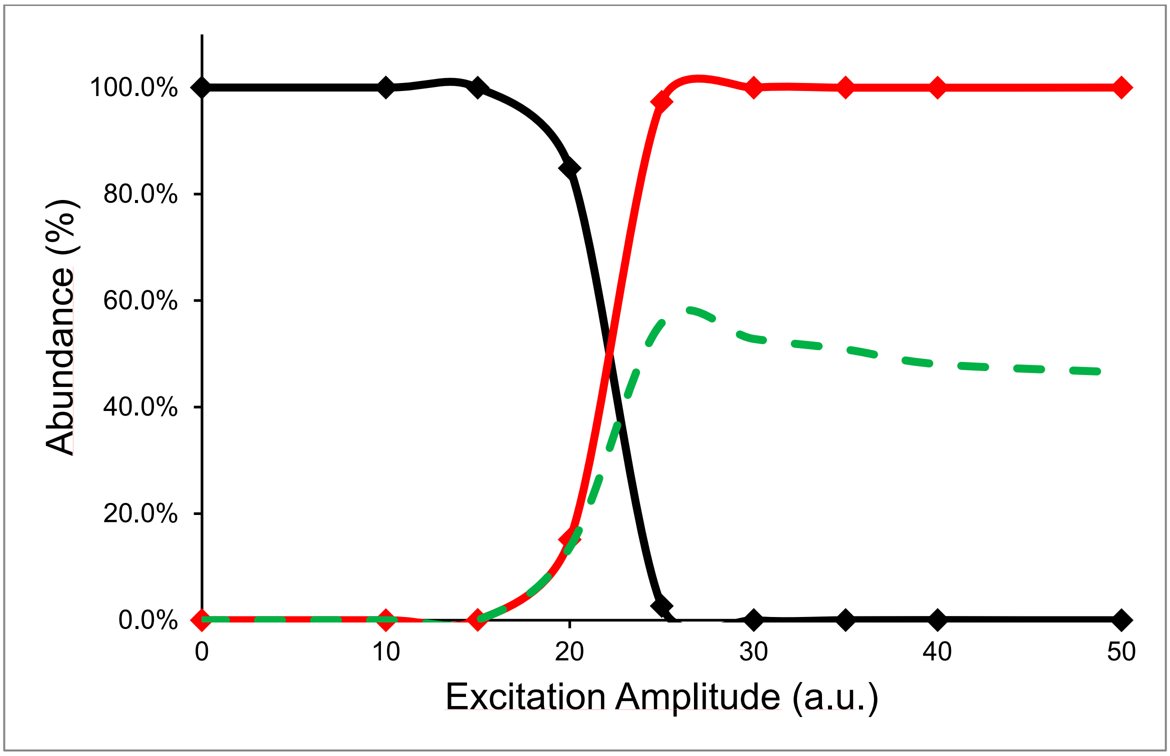

Figure S1. Precursor SY curve (black line), PiF (red line) and PiY (green dashed line) of the 3-CQA isomer at ExT 50 ms.

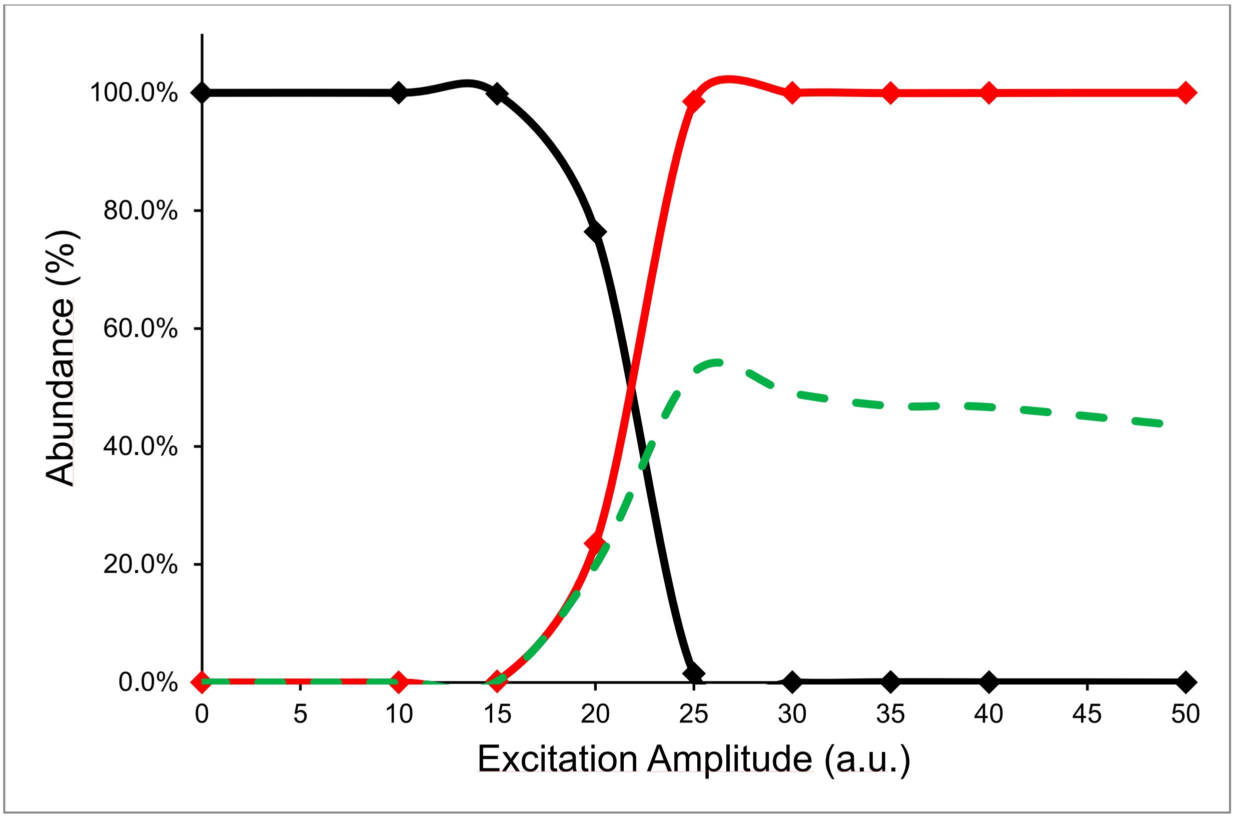

Figure S2. Precursor SY curve (black line), PiF (red line) and PiY (green dashed line) of the 4-CQA isomer at ExT 50 ms.

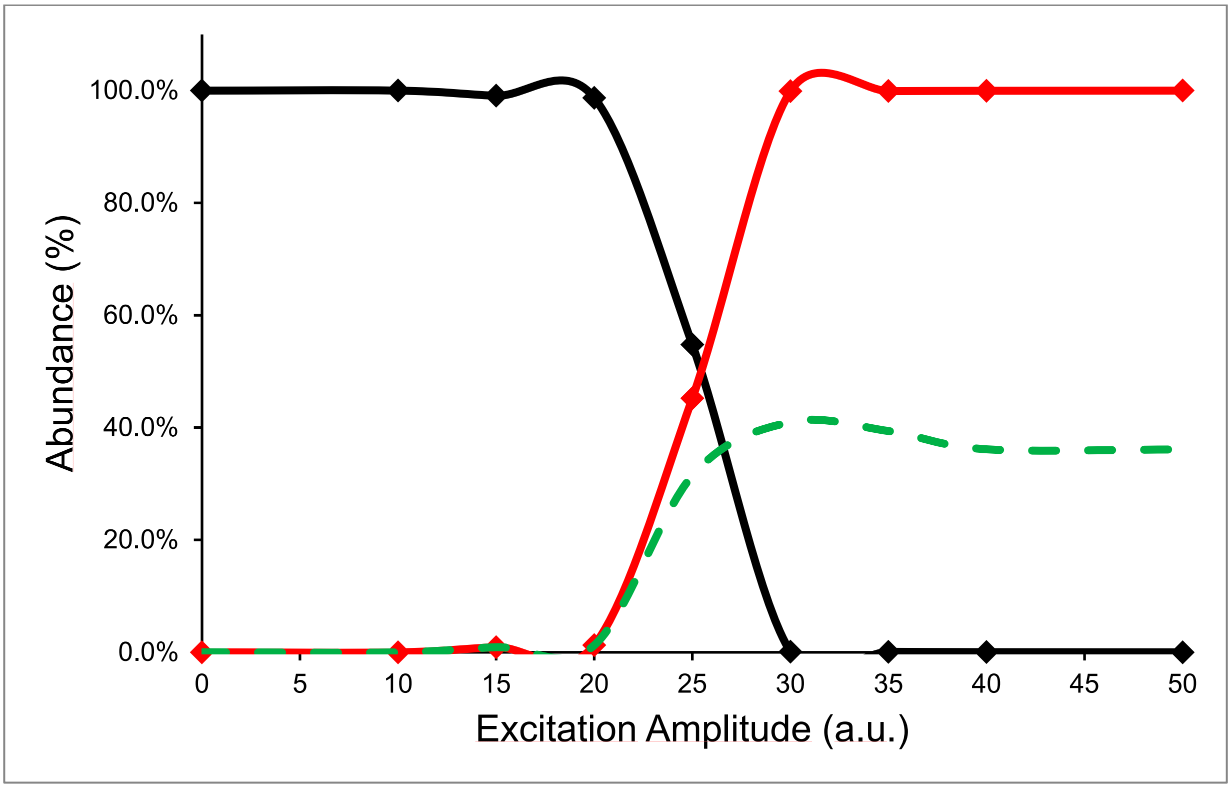

Figure S3. Precursor SY curve (black line), PiF (red line) and PiY (green dashed line) of the 3,4-diCQA isomer at ExT 50 ms.

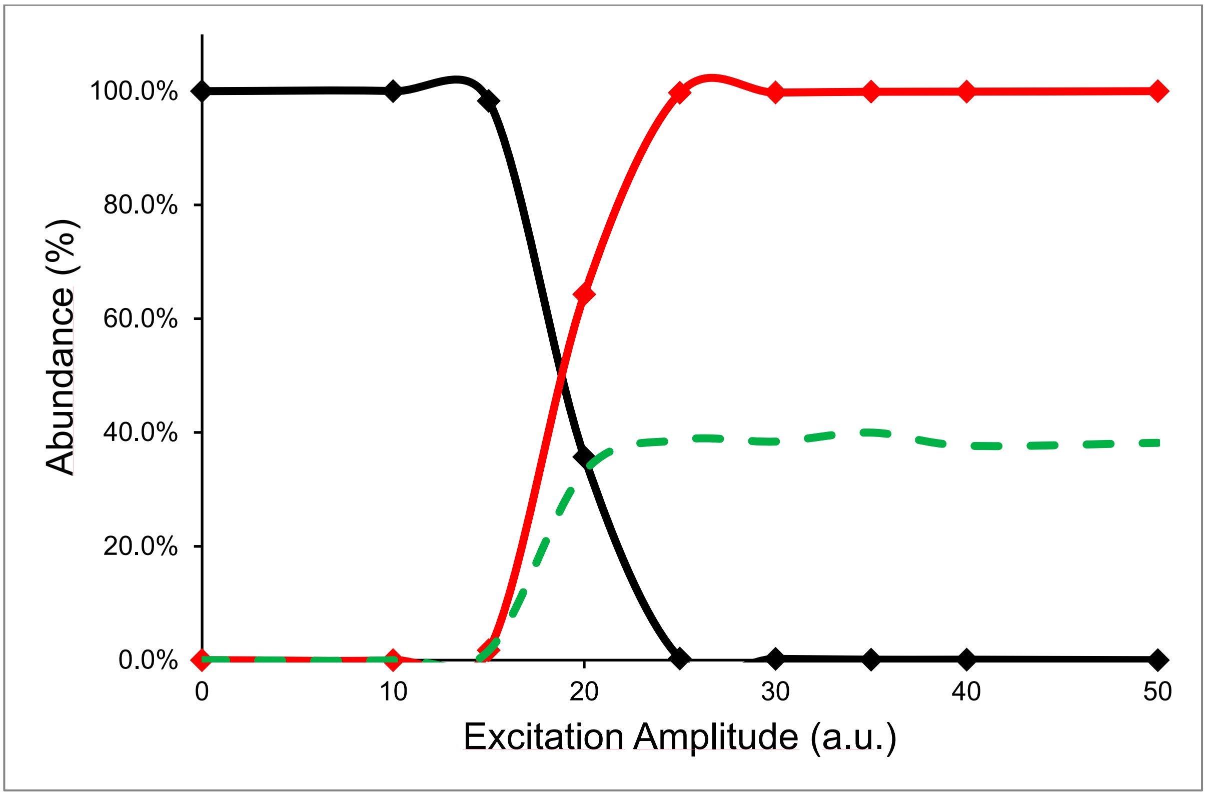

Figure S4. Precursor SY curve (black line), PiF (red line) and PiY (green dashed line) of the 3,5-diCQA isomer at ExT 50 ms.

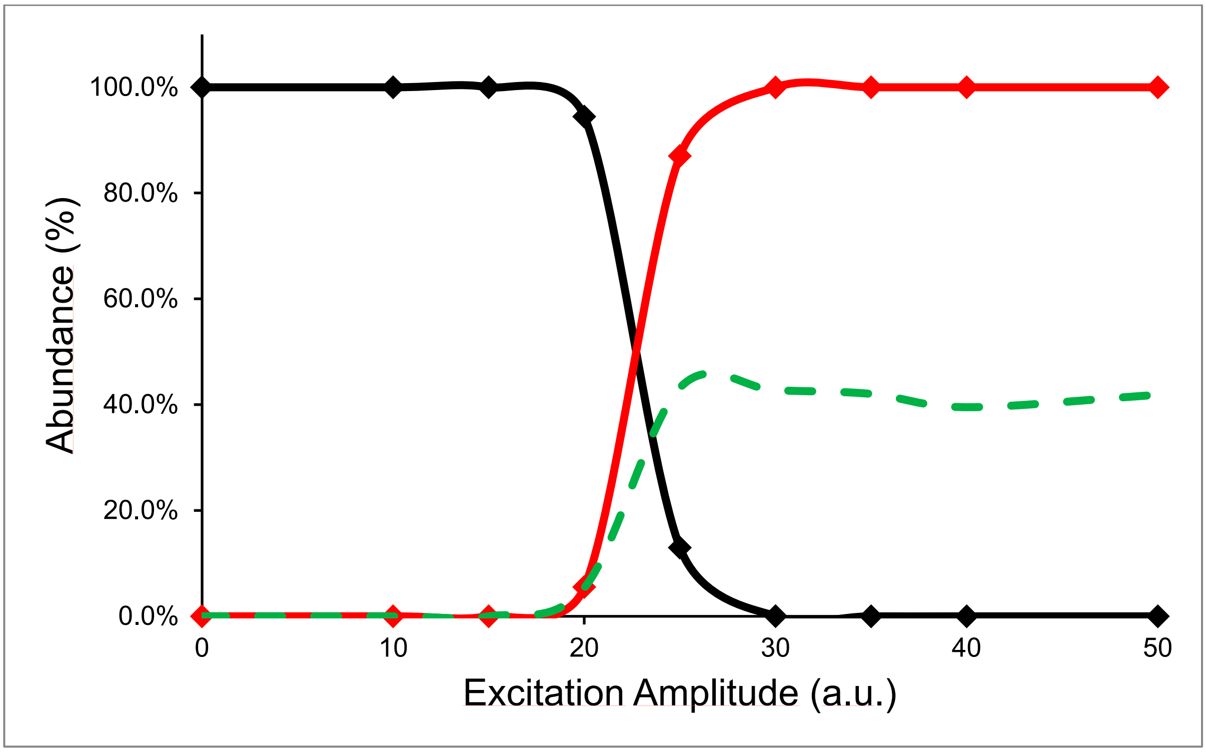

Figure S5. Precursor SY curve (black line), PiF (red line) and PiY (green dashed line) of the 4,5-diCQA isomer at ExT 50 ms.

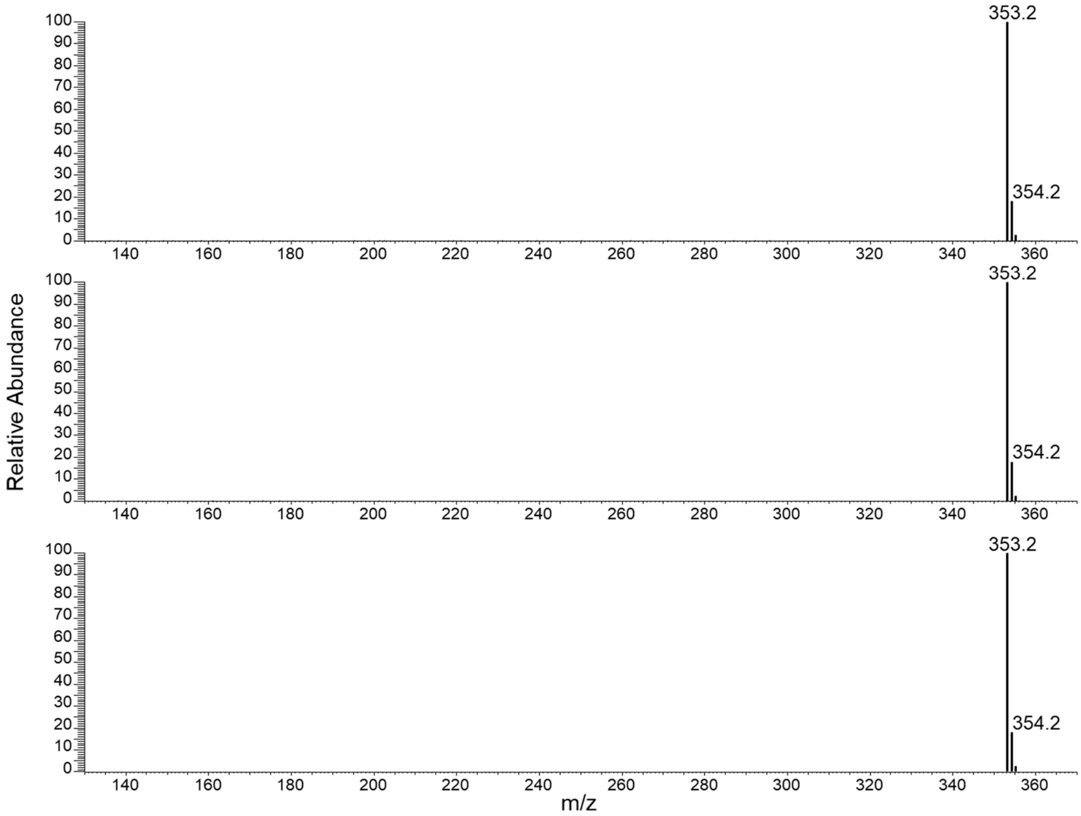

Figure S6. ESI negative ions spectra of 3-CQA (upper), 5-CQA (middle) and 4-CQA (bottom).

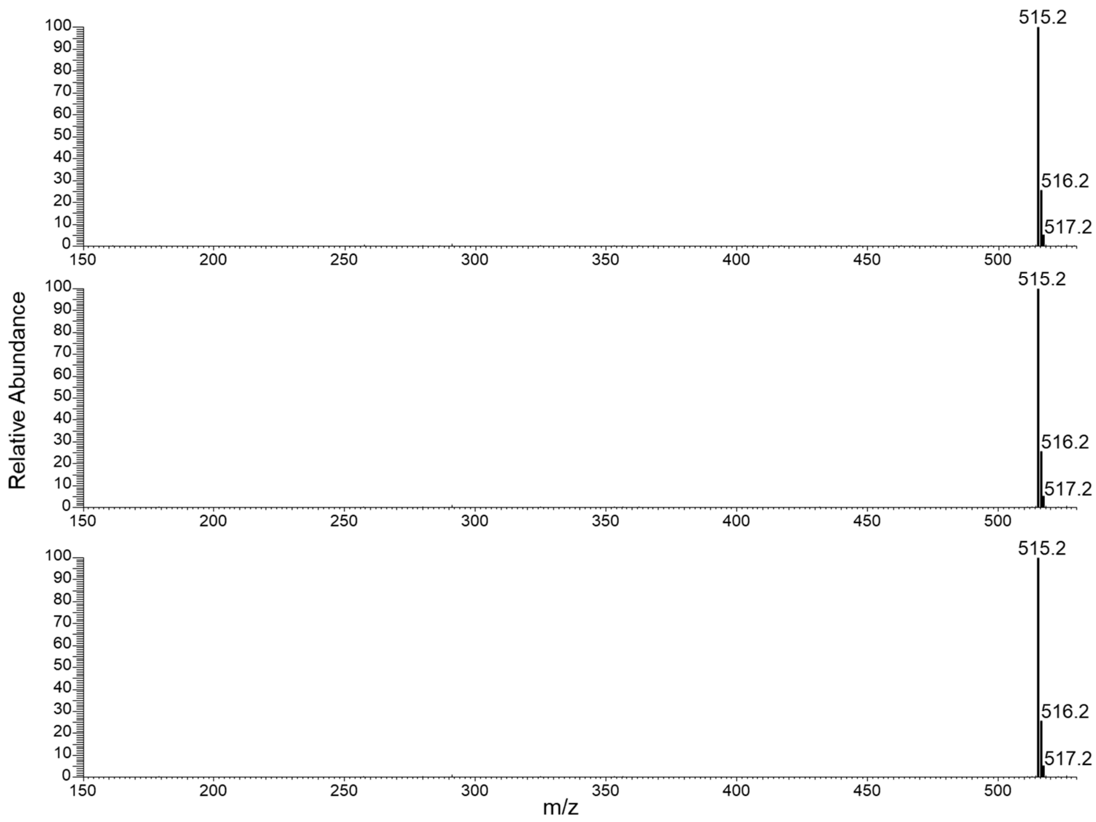

Figure S7. ESI negative ions spectra of 3,4-diCQA (upper), 3,5-diCQA (middle) and 4,5-diCQA (bottom).

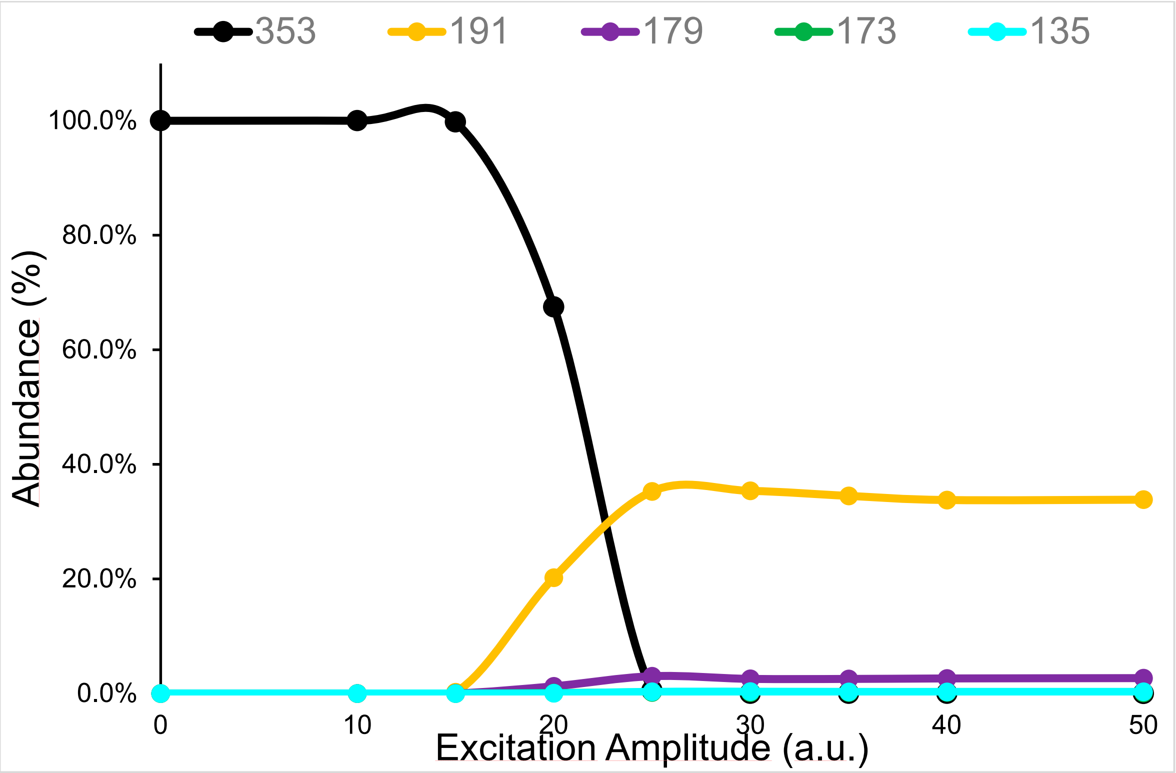

Figure S8. The breakdown curves of 5-CQA isomer obtained by elaboration of ERMS data.

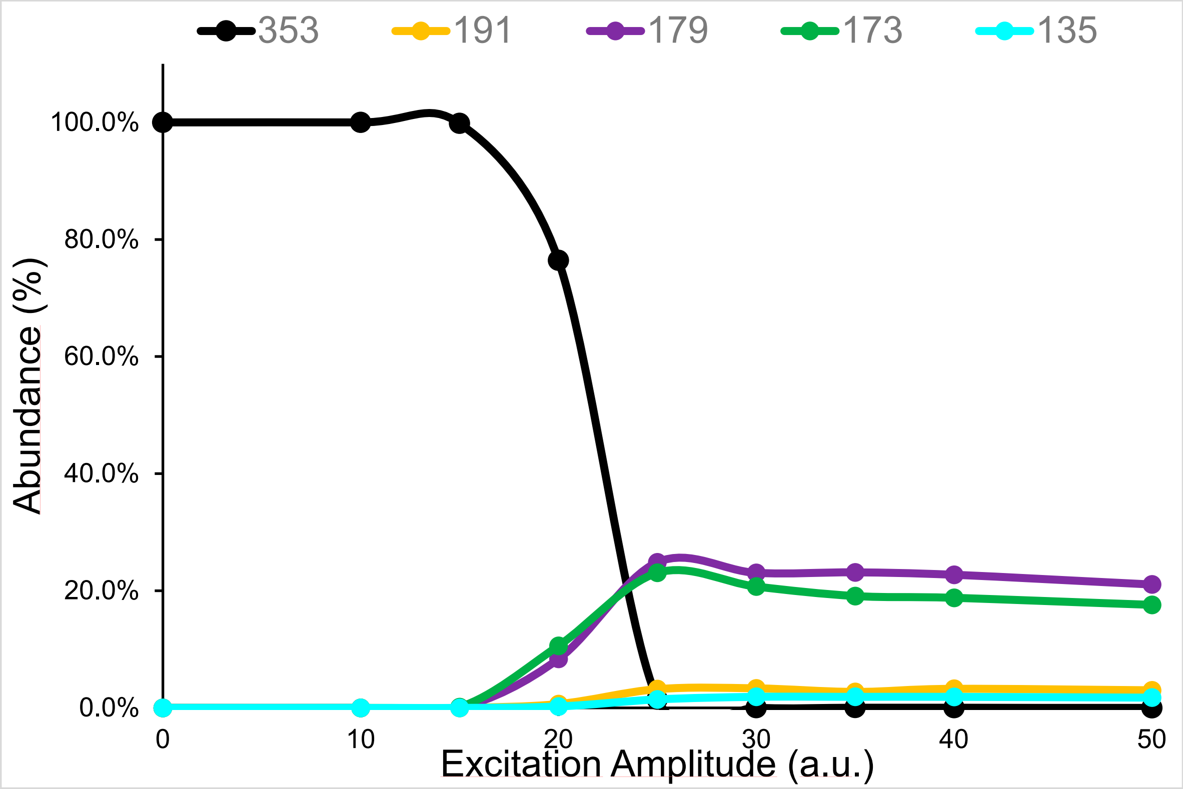

Figure S9. The breakdown curves of 4-CQA isomer obtained by elaboration of ERMS data.

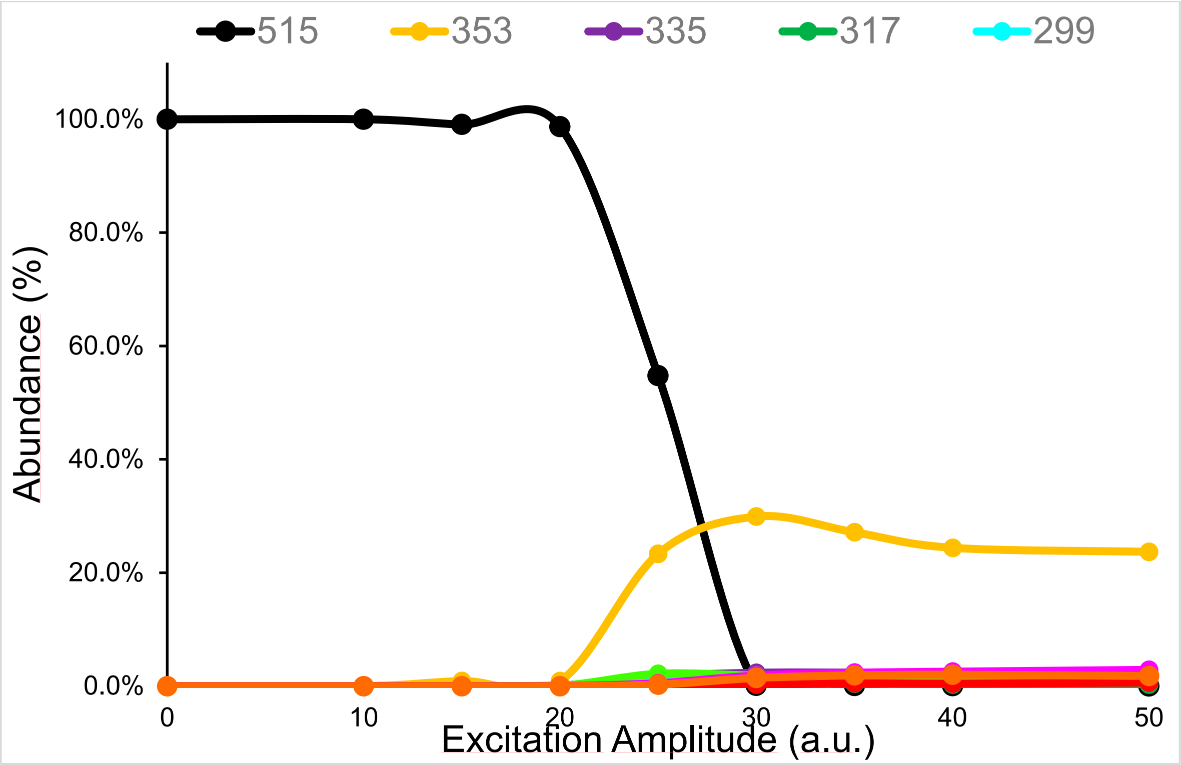

Figure S10. The breakdown curves of 3,4-diCQA isomer obtained by elaboration of ERMS data.

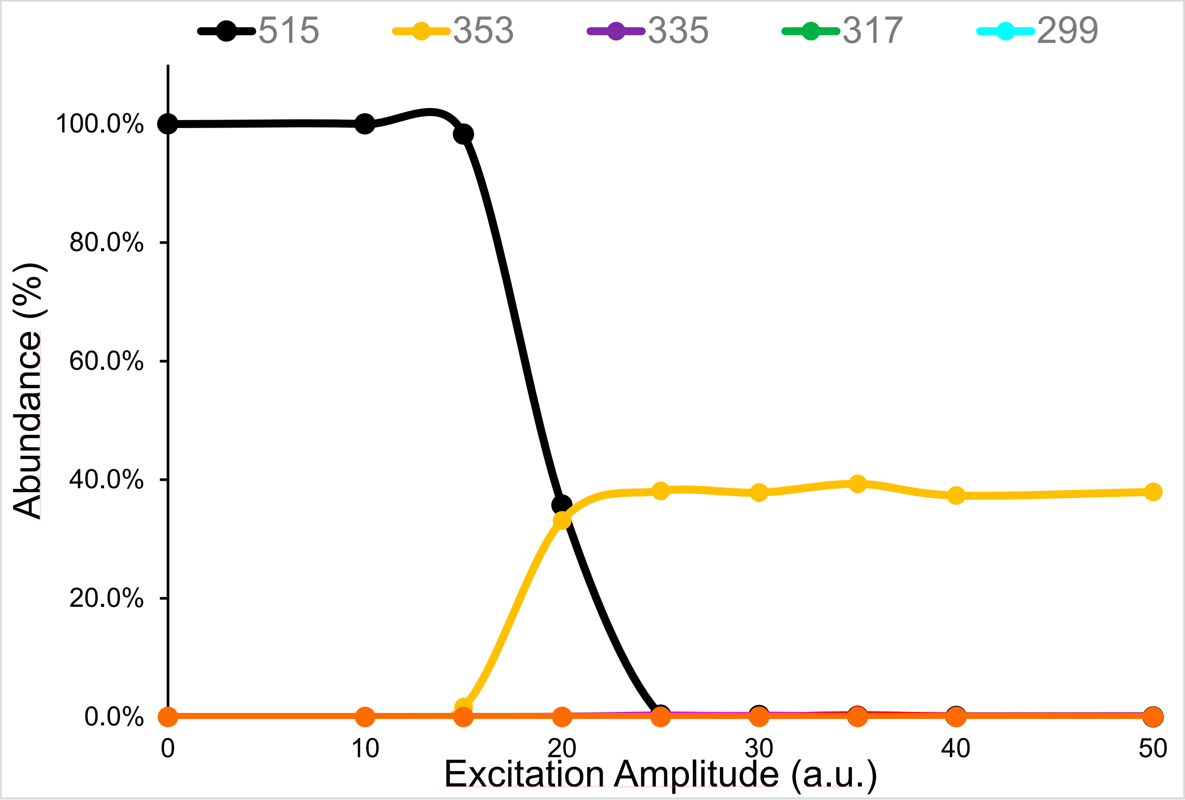

Figure S11. The breakdown curves of 3,5-diCQA isomer obtained by elaboration of ERMS data.

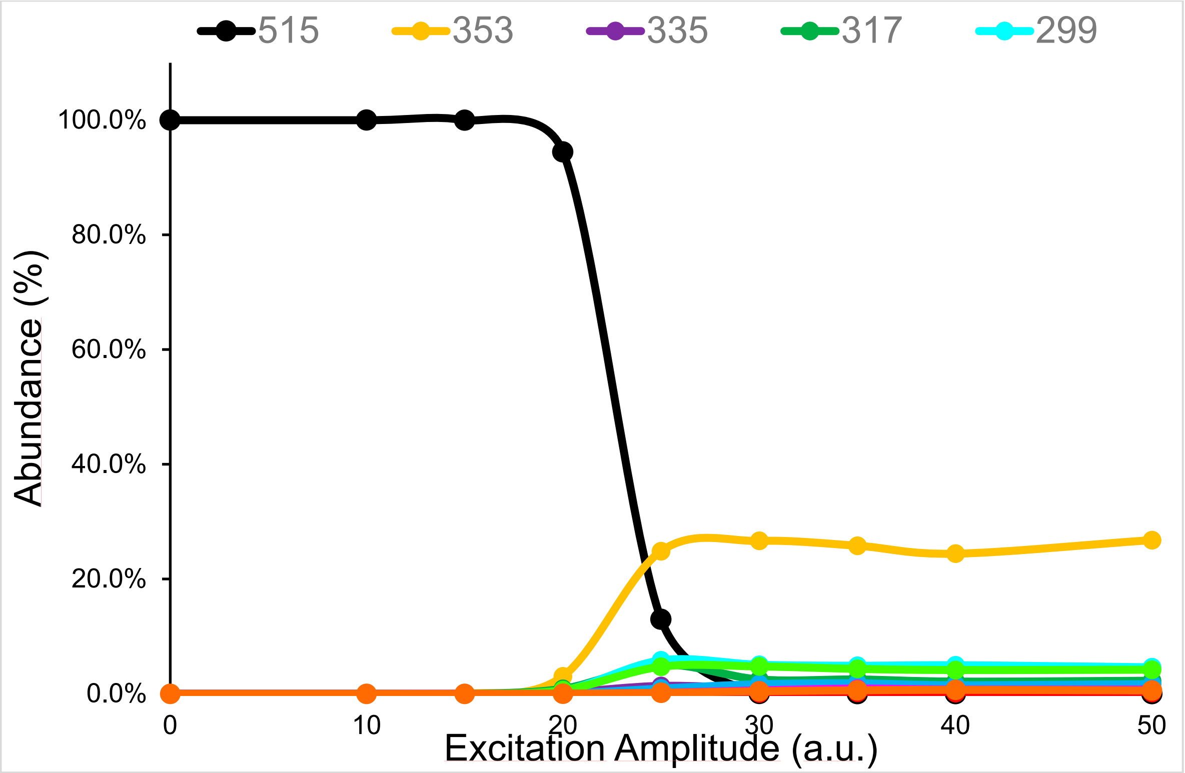

Figure S12. The breakdown curves of 4,5-diCQA isomer obtained by elaboration of ERMS data.

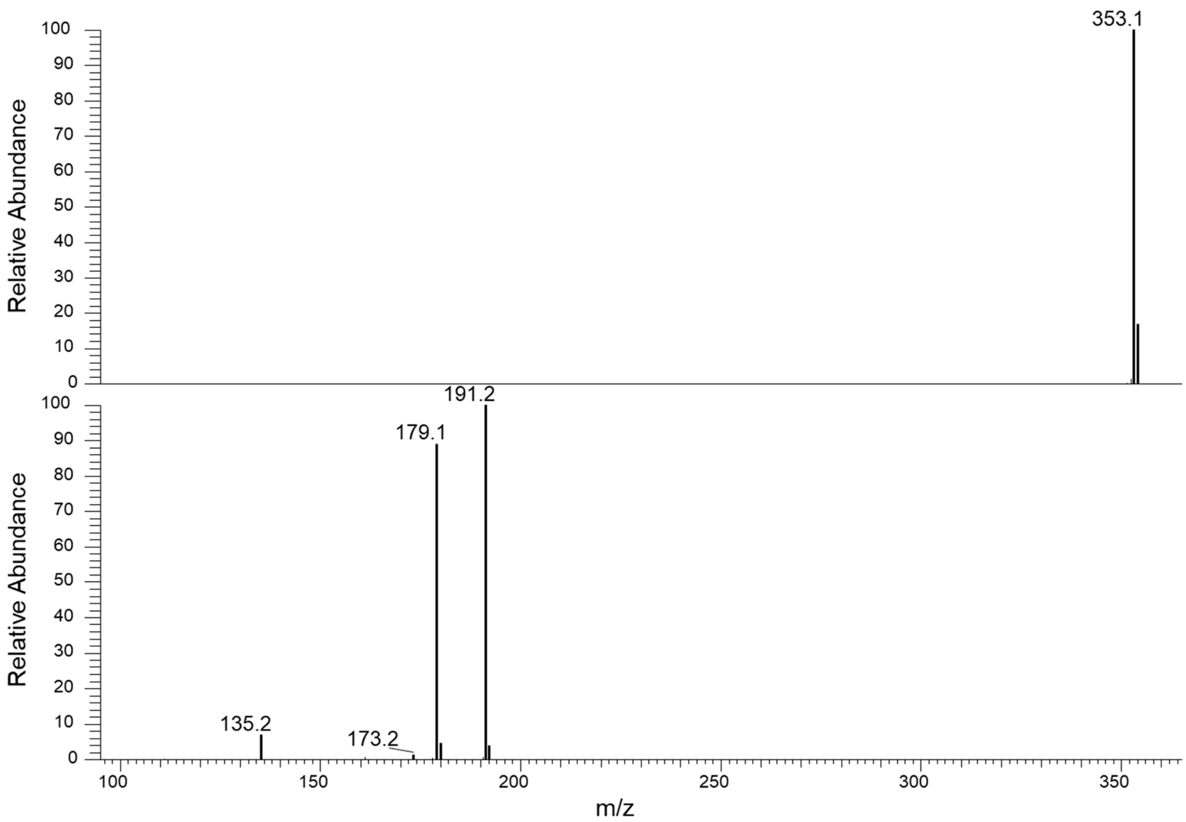

Figure S13. Comparison of the MS/MS spectra obtained at low ExA (MS/MS event 1, above), used to monitor the Ri signal, and at high ExA (MS/MS event 2, below), to acquire the Pis signals of 3-CQA.

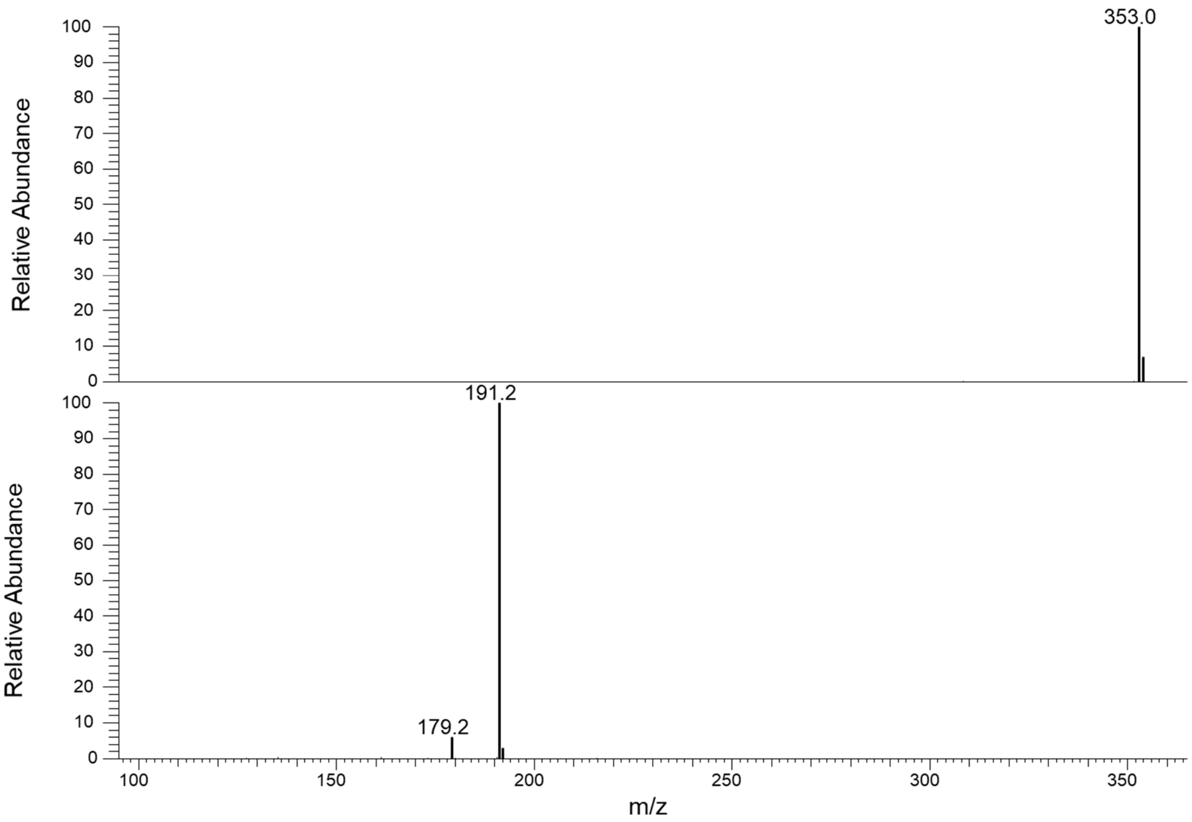

Figure S14. Comparison of the MS/MS spectra obtained at low ExA (MS/MS event 1, above), used to monitor the Ri signal, and at high ExA (MS/MS event 2, below), to acquire the Pis signals of 5-CQA.

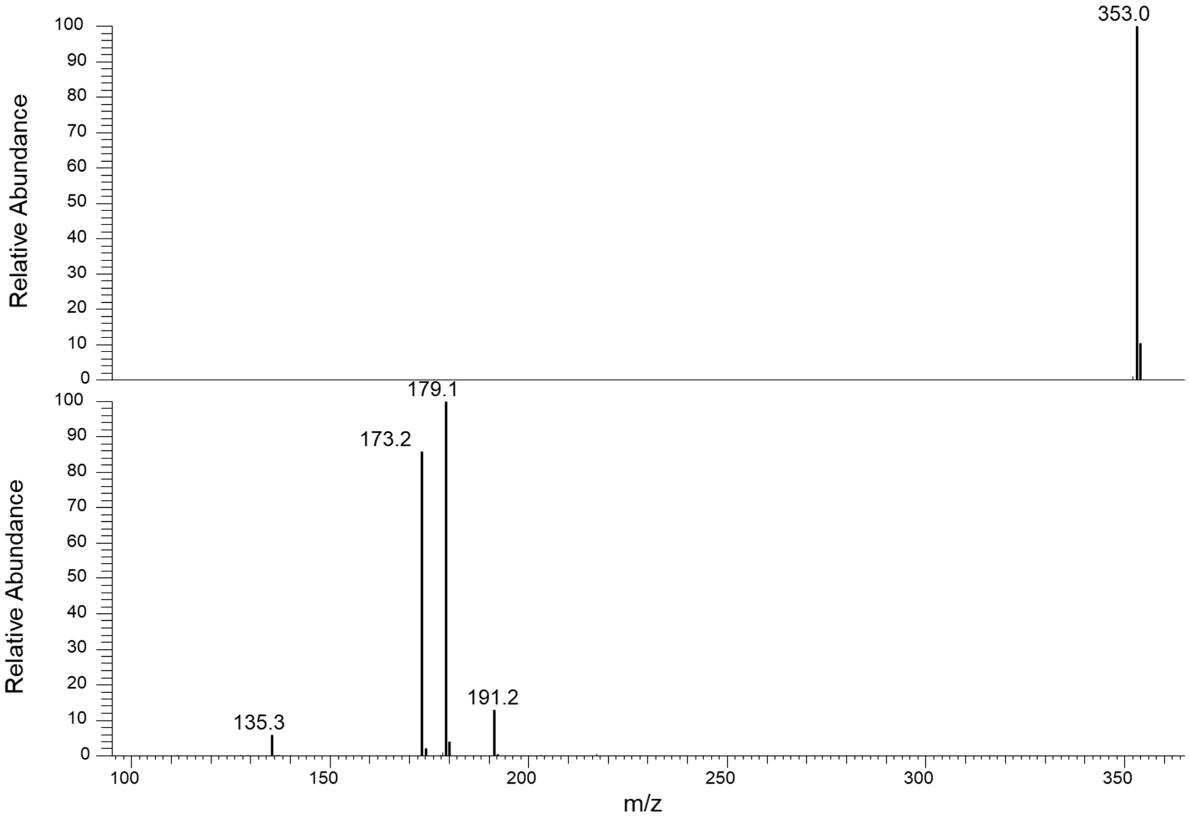

Figure S15. Comparison of the MS/MS spectra obtained at low ExA (MS/MS event 1, above), used to monitor the Ri signal, and at high ExA (MS/MS event 2, below), to acquire the Pis signals of 4-CQA.

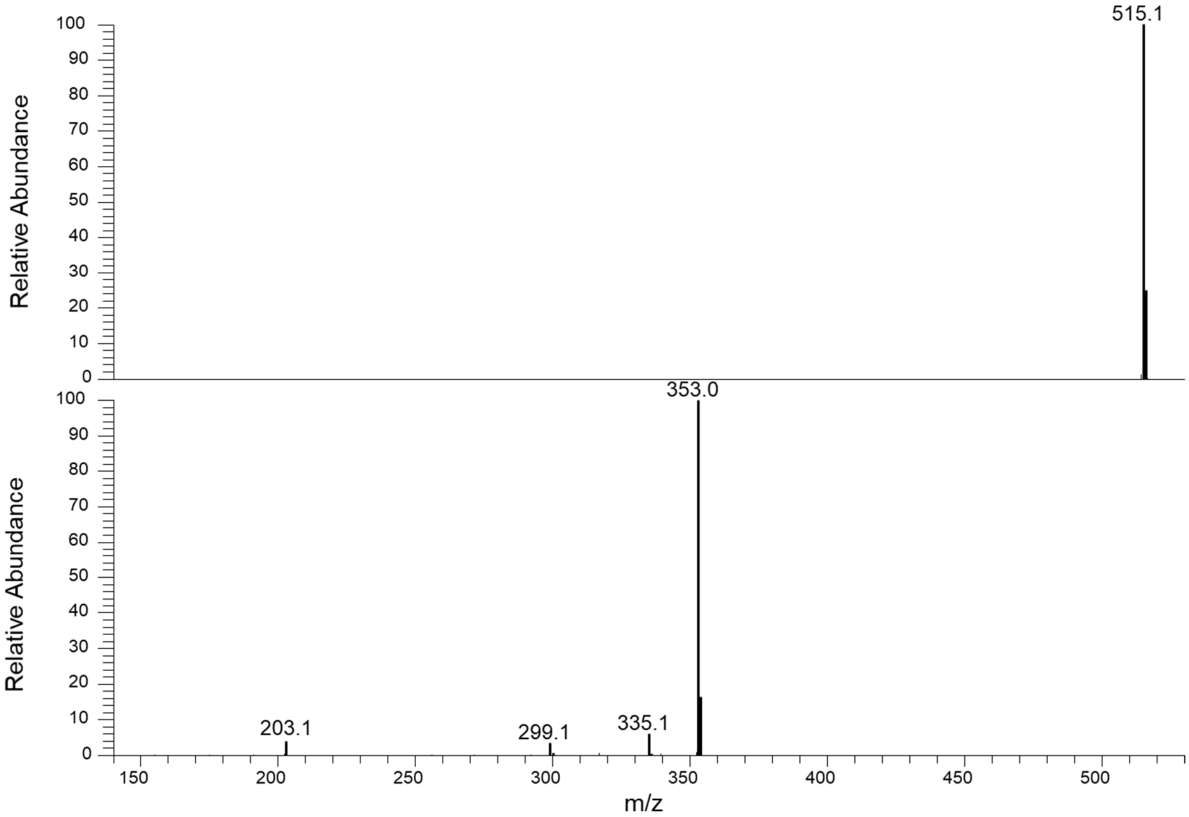

Figure S16. Comparison of the MS/MS spectra obtained at low ExA (MS/MS event 1, above), used to monitor the Ri signal, and at high ExA (MS/MS event 2, below), to acquire the Pis signals of 3,4-diCQA.

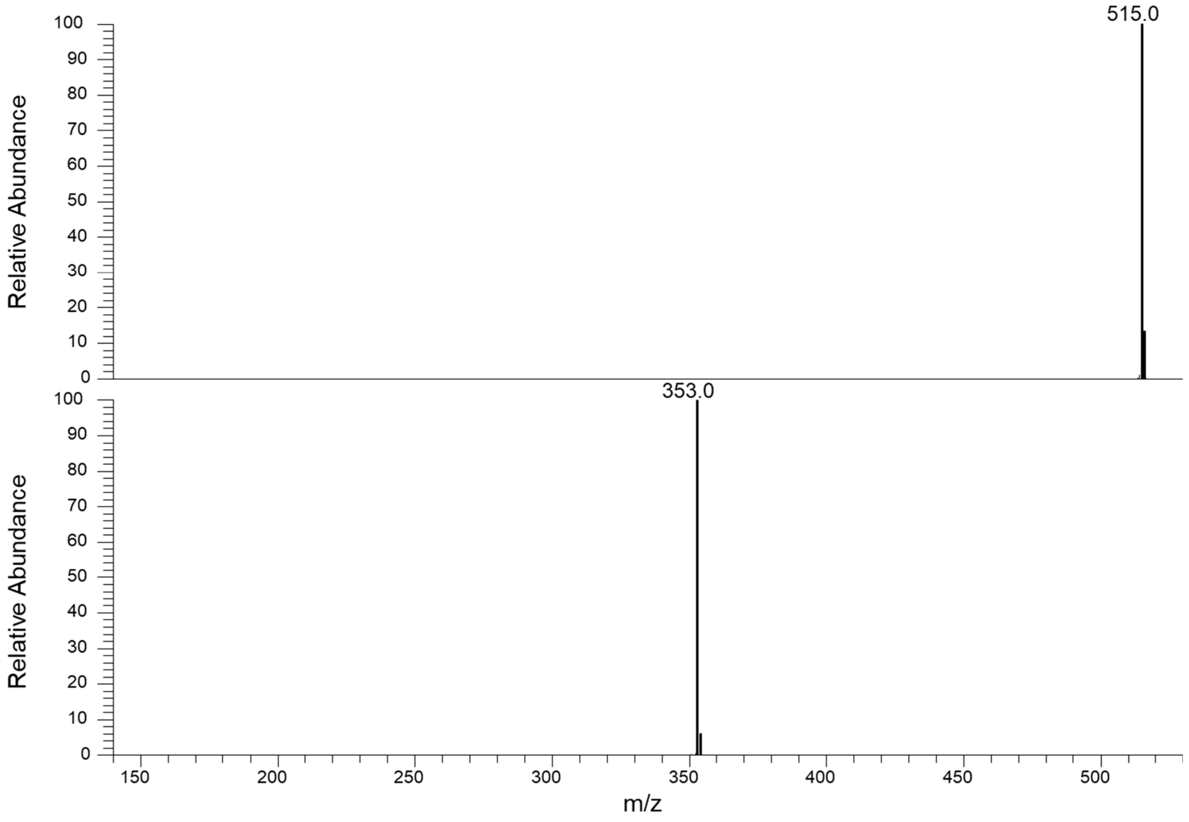

Figure S17. Comparison of the MS/MS spectra obtained at low ExA (MS/MS event 1, above), used to monitor the Ri signal, and at high ExA (MS/MS event 2, below), to acquire the Pis signals of 3,5-diCQA.

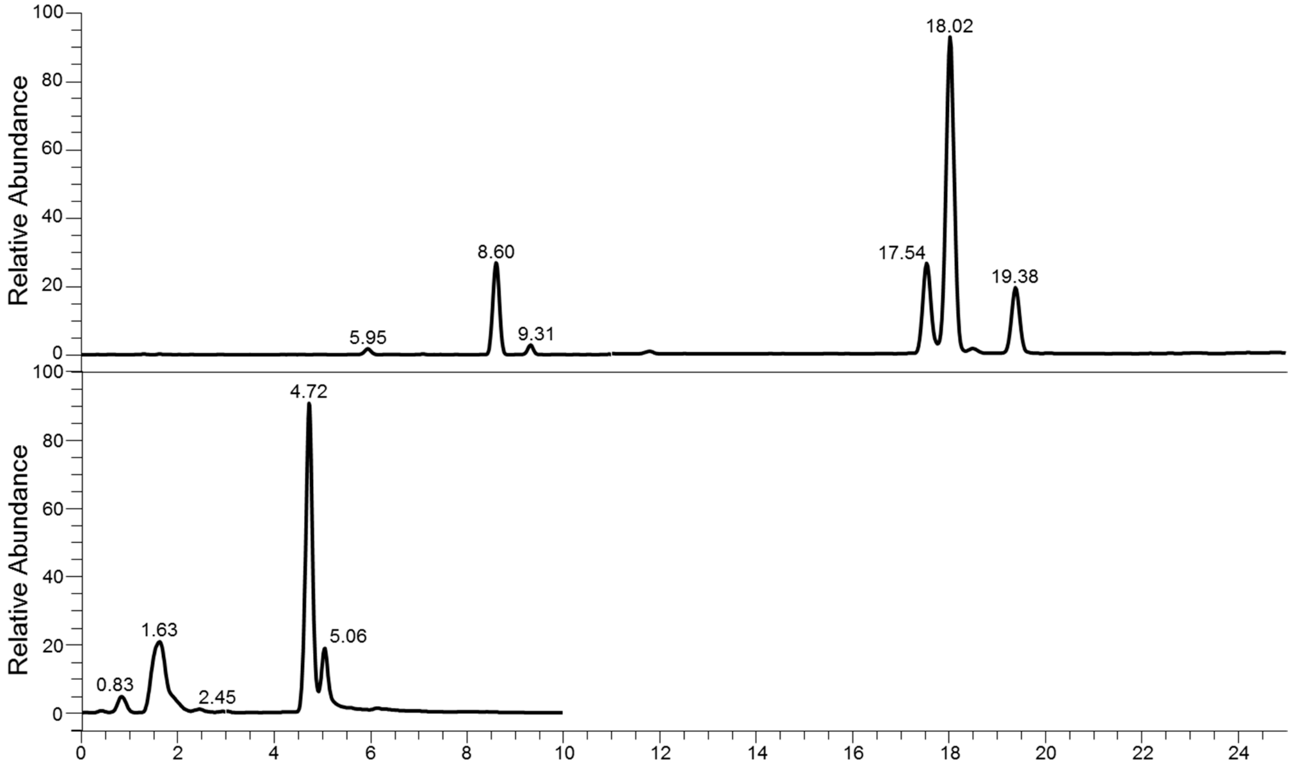

Figure S18. Comparison between the HLPC-MS/MS analysis of Acmella WS with ChromSys 1 (above) and ChromSys 2 (bottom).

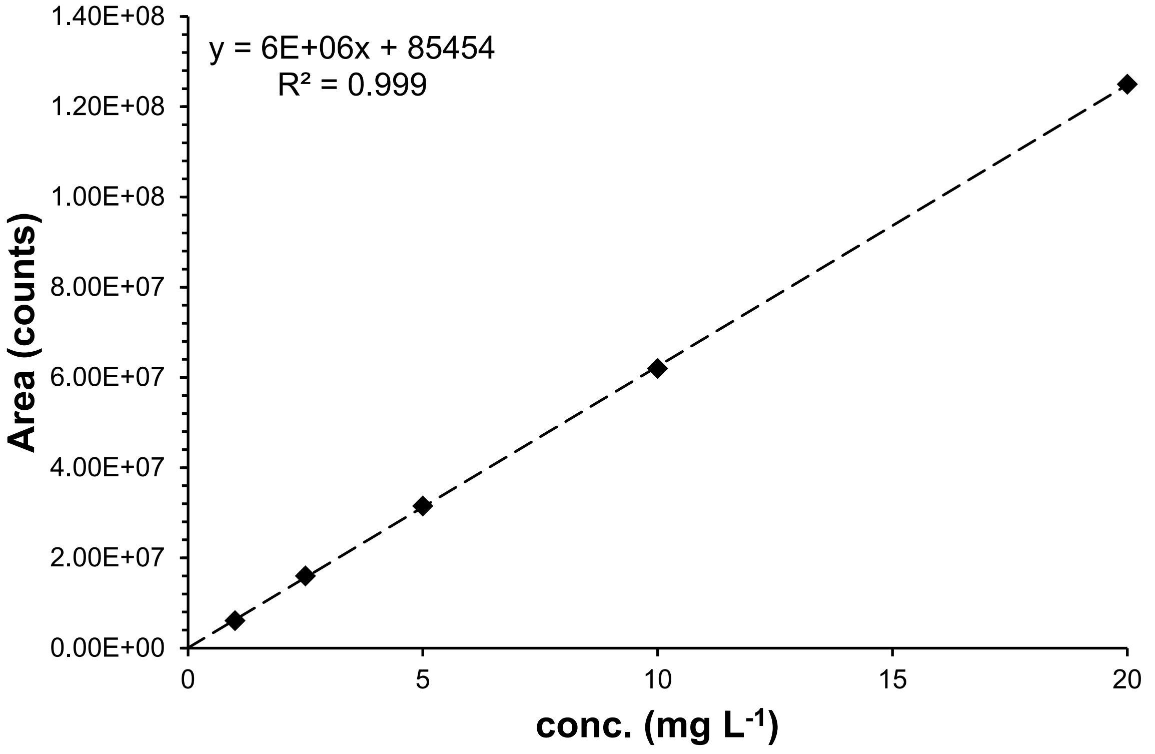

Figure S19. The calibration curve of 5-CQA.

LEDA algorithm

Equation S1. LEDA$_{CQA}$ matrix for CQAs isomers speciation:

$$\begin{bmatrix} Area_{191}/Area_{Ri} = \left({}^{Pi_{191}}/_{Ri}\right)_{isomer1}[\%]_{isomer1} + \left({}^{Pi_{1191}}/_{Ri}\right)_{isomer2}[\%]_{isomer2} + \left({}^{Pi_{191}}/_{Ri}\right)_{isomer3}[\%]_{isomer3} \\ Area_{179}/Area_{Ri} = \left({}^{Pi_{179}}/_{Ri}\right)_{isomer1}[\%]_{isomer1} + \left({}^{Pi_{179}}/_{Ri}\right)_{isomer2}[\%]_{isomer2} + \left({}^{Pi_{179}}/_{Ri}\right)_{isomer3}[\%]_{isomer3} \\ Area_{173}/Area_{Ri} = \left({}^{Pi_{173}}/_{Ri}\right)_{isomer1}[\%]_{isomer1} + \left({}^{Pi_{173}}/_{Ri}\right)_{isomer2}[\%]_{isomer2} + \left({}^{Pi_{173}}/_{Ri}\right)_{isomer3}[\%]_{isomer3} \\ Area_{135}/Area_{Ri} = \left({}^{Pi_{135}}/_{Ri}\right)_{isomer1}[\%]_{isomer1} + \left({}^{Pi_{135}}/_{Ri}\right)_{isomer2}[\%]_{isomer2} + \left({}^{Pi_{135}}/_{Ri}\right)_{isomer3}[\%]_{isomer3} \end{bmatrix}$$

$$\begin{bmatrix} Area_{191}/Area_{Ri} \\ Area_{179}/Area_{Ri} \\ Area_{173}/Area_{Ri} \\ Area_{135}/Area_{Ri} \end{bmatrix} = A$$

$$\begin{bmatrix} \left({}^{Pi_{191}}/_{Ri}\right)_{isomer1} & \left({}^{Pi_{191}}/_{Ri}\right)_{isomer2} & \left({}^{Pi_{191}}/_{Ri}\right)_{isomer\ 3} \\ \left({}^{Pi_{179}}/_{Ri}\right)_{isomer1} & \left({}^{Pi_{179}}/_{Ri}\right)_{isomer2} & \left({}^{Pi_{179}}/_{Ri}\right)_{isomer3} \\ \left({}^{Pi_{173}}/_{Ri}\right)_{isomer1} & \left({}^{Pi_{173}}/_{Ri}\right)_{isomer2} & \left({}^{Pi_{173}}/_{Ri}\right)_{isomer3} \\ \left({}^{Pi_{135}}/_{Ri}\right)_{isomer1} & \left({}^{Pi_{135}}/_{Ri}\right)_{isomer2} & \left({}^{Pi_{135}}/_{Ri}\right)_{isomer3} \end{bmatrix} = K$$

$$\left[[\%]_{Isomer1} \quad [\%]_{isomer2} \quad [\%]_{isomer3}\right] = X$$

Equation S2. LEDA$_{diCQA}$ matrix for diCQAs isomers speciation:

$$\begin{bmatrix} Area_{353}/Area_{Ri} = \left({}^{Pi_{353}}/_{Ri}\right)_{isomer1}[\%]_{isomer1} + \left({}^{Pi_{2033}}/_{Ri}\right)_{isomer2}[\%]_{isomer2} + \left({}^{Pi_{353}}/_{Ri}\right)_{isomer3}[\%]_{isomer3} \\ Area_{335}/Area_{Ri} = \left({}^{Pi_{335}}/_{Ri}\right)_{isomer1}[\%]_{isomer1} + \left({}^{Pi_{335}}/_{Ri}\right)_{isomer2}[\%]_{isomer2} + \left({}^{Pi_{335}}/_{Ri}\right)_{isomer3}[\%]_{isomer3} \\ Area_{317}/Area_{Ri} = \left({}^{Pi_{317}}/_{Ri}\right)_{isomer1}[\%]_{isomer1} + \left({}^{Pi_{317}}/_{Ri}\right)_{isomer2}[\%]_{isomer2} + \left({}^{Pi_{317}}/_{Ri}\right)_{isomer3}[\%]_{isomer3} \\ Area_{203}/Area_{Ri} = \left({}^{Pi_{203}}/_{Ri}\right)_{isomer1}[\%]_{isomer1} + \left({}^{Pi_{203}}/_{Ri}\right)_{isomer2}[\%]_{isomer2} + \left({}^{Pi_{203}}/_{Ri}\right)_{isomer3}[\%]_{isomer3} \\ Area_{203}/Area_{Ri} = \left({}^{Pi_{203}}/_{Ri}\right)_{isomer1}[\%]_{isomer1} + \left({}^{Pi_{203}}/_{Ri}\right)_{isomer2}[\%]_{isomer2} + \left({}^{Pi_{203}}/_{Ri}\right)_{isomer3}[\%]_{isomer3} \end{bmatrix}$$

$$\begin{bmatrix} Area_{353}/Area_{Ri} \\ Area_{335}/Area_{Ri} \\ Area_{317}/Area_{Ri} \\ Area_{299}/Area_{Ri} \\ Area_{203}/Area_{Ri} \end{bmatrix} = A$$

$$\begin{bmatrix} \left({Pi_{353}}/{Ri}\right)_{isomer1} & \left({Pi_{353}}/{Ri}\right)_{isomer2} & \left({Pi_{353}}/{Ri}\right)_{isomer\ 3} \\ \left({Pi_{335}}/{Ri}\right)_{isomer1} & \left({Pi_{335}}/{Ri}\right)_{isomer2} & \left({Pi_{335}}/{Ri}\right)_{isomer\ 3} \\ \left({Pi_{317}}/{Ri}\right)_{isomer1} & \left({Pi_{317}}/{Ri}\right)_{isomer2} & \left({Pi_{317}}/{Ri}\right)_{isomer3} \\ \left({Pi_{299}}/{Ri}\right)_{isomer1} & \left({Pi_{299}}/{Ri}\right)_{isomer2} & \left({Pi_{299}}/{Ri}\right)_{isomer3} \\ \left({Pi_{203}}/{Ri}\right)_{isomer1} & \left({Pi_{203}}/{Ri}\right)_{isomer2} & \left({Pi_{203}}/{Ri}\right)_{isomer3} \end{bmatrix} = K$$

$$[[\%]_{Isomer1} \quad [\%]_{isomer2} \quad [\%]_{isomer3}] = X$$

A general equation to solve each overdetermined LEDA matrix:

Traspose K matrix = K'

Inverse $(K \times K')$ = $(K \times K')^{-1}$

$$X = (A \times K') \times (K \times K')^{-1} \qquad \textbf{(Equation S3)}$$
